# Pedicle screw loosening after degenerative lumbar fusion: a systematic review and meta-analysis of preoperative imaging-derived bone quality and paraspinal muscle metrics

**DOI:** 10.1007/s00701-026-06984-3

**Published:** 2026-07-30

**Authors:** Shaan Patel, Shiva A. Nischal, Lorenzo Ceccon, Gargee Sree Nallanukala, Kush M. Kale, Advith Sarikonda, Stephanie Serva, Jack Jallo, James S. Harrop, Srinivas K. Prasad

**Affiliations:** 1 Department of Neurological Surgery, Thomas Jefferson University Hospital, Philadelphia, PA USA; 2 Department of Physiology, Anatomy & Genetics, Medical Sciences Division, University of Oxford, Oxford, UK; 3 Philadelphia College of Osteopathic Medicine, Philadelphia, PA USA

**Keywords:** Pedicle screw loosening, Lumbar fusion, Bone quality, Hounsfield units, Vertebral bone quality, Diagnostic accuracy, Risk stratification

## Abstract

**Background:**

Radiographic pedicle screw loosening (PSL) remains a frequent postoperative finding and potential marker of screw-bone interface compromise after degenerative lumbar fusion, yet preoperative risk stratification remains inconsistent. This systematic review and meta-analysis aims to compare the association and discriminative performance of preoperative imaging-derived bone quality and paraspinal muscle metrics for predicting radiographic PSL after degenerative lumbar fusion.

**Methods:**

PubMed, Embase, and CENTRAL were searched until January 2026. Observational studies reporting radiographic PSL and evaluating preoperative imaging-based bone or muscle metrics were included. Bone metrics comprised CT-derived Hounsfield units (HU), CT-derived bone mineral density (CT-BMD), MRI-based vertebral bone quality (VBQ), and DEXA T-scores. Muscle measures included psoas and posterior paraspinal cross-sectional area (CSA) and composition. Radiographic PSL definitions and adjudication modalities (plain radiograph versus CT) varied across included studies and were considered explicitly during synthesis and interpretation. Continuous outcomes were synthesized using random-effects inverse-variance models. Diagnostic accuracy was evaluated using bivariate (Reitsma) generalized linear mixed models, with summary receiver operating characteristic (SROC)-derived area under the curve (AUC) used to quantify discrimination. Risk of bias was assessed using QUADAS-2.

**Results:**

Twenty-nine studies (4711 patients; 25.4% PSL) were included. Patients with PSL demonstrated poorer preoperative bone quality across modalities, including lower HU (MD -24.7; *P* < 0.01), lower CT-BMD (MD -28.05; *P* < 0.05), lower DEXA T-scores (MD -0.18; *P* < 0.01), higher VBQ (MD 0.46; *P* < 0.01). Diagnostic discrimination was moderate and comparable across metrics (SROC-derived pooled AUC 0.71–0.76; meta-regression *P* = 0.08), although pooled estimates reflect a composite radiographic endpoint with heterogeneous adjudication conventions rather than a single uniform reference standard. Among muscle measures, reduced psoas CSA showed consistent association with PSL, whereas posterior paraspinal metrics were heterogeneous and largely non-significant. Nomogram analyses demonstrated clinically meaningful risk reclassification but limited rule-in and rule-out capacity.

**Conclusions:**

Imaging-derived bone quality metrics identify substrate-level vulnerability to radiographic PSL and provide moderate preoperative discrimination. Because pooled discrimination reflects a composite radiographic endpoint rather than a uniform reference standard, these metrics are moderate risk stratifiers for radiographic PSL, not validated stand-alone tests for clinically meaningful construct failure. Limited and heterogeneous evidence suggests psoas size may represent a complementary frailty marker. Prospective validation and standardized thresholds are required.

**Supplementary Information:**

The online version contains supplementary material available at https://doi.org/10.1007/s00701-026-06984-3.

## Introduction

Pedicle screw fixation is fundamental to the stability of modern lumbar fusion constructs, providing resistance to translational, rotational, and bending forces during the fusion process. Despite advances in implant design and surgical technique, pedicle screw loosening (PSL) remains a frequent mode of delayed instrumentation failure, particularly in degenerative lumbar disease. Reported incidence varies widely, reflecting differences in patient selection, construct design, surveillance strategy, and diagnostic thresholds, but radiographic loosening is consistently associated with inferior mechanical stability and adverse clinical sequelae [72, 73].

Unlike acute implant failure, PSL is characteristically a progressive, time-dependent phenomenon [72]. Radiographic halo formation or peri-screw radiolucency represents the end stage of a process driven by repetitive cyclic loading at the screw-bone interface [2]. Even when initial fixation is satisfactory, small degrees of micro-motion can accumulate over time, leading to trabecular resorption, loss of interfacial stiffness, and eventual compromise of construct integrity [30, 52, 72]. From a clinician’s perspective, the problem is most often first recognized not as a “mode of failure,” but as an imaging finding: peri-screw radiolucency (“halo” or “double halo” sign) on routine postoperative radiographs or CT. The clinical meaning of this lucency is frequently uncertain at the point of detection, ranging from a stable interface change without clinical consequence to a radiographic signature of progressive screw-bone interface failure that may precede pseudarthrosis, recurrent symptoms, loss of correction, or revision [2, 15, 73]. Interpretation is further complicated by variability across studies in how lucency/PSL is defined and which modality is used to adjudicate loosening (plain radiographs versus CT). In this context, it is important to distinguish PSL from clinically significant construct failure. In modern practice, PSL is typically defined as a peri-screw radiolucent zone (≥ 1 mm), halo or double-halo sign, or progressive circumferential lucency on follow-up imaging, irrespective of symptom status. While these findings may precede mechanical instability, pseudoarthrosis, or clinical deterioration, they do not uniformly indicate construct failure. Accordingly, PSL is conceptualized here as a radiographic marker of interface-level vulnerability rather than a direct surrogate for symptomatic failure. Within the Panjabi functional spinal unit, the screw-bone interface is only one component of construct stability; the prediction target here is therefore radiographic PSL specifically (peri-screw lucency, halo or double-halo formation, or progressive circumferential radiolucency), not fixation-fatigue endpoints such as pseudarthrosis, loss of correction, or revision. Reduced preoperative Hounsfield unit or VBQ values thus mark a lower-tolerance interface even when they do not mandate revision. This conceptual distinction informed our analytic plan: eligibility was restricted to cohorts adjudicating PSL radiographically, clinical and fusion outcomes treated as secondary descriptors, and pooled estimates are interpreted as reflecting substrate-level interface vulnerability rather than symptomatic failure. Heterogeneity in surveillance intensity, imaging modality, and clinical context is acknowledged throughout. From a mechanistic perspective, screw loosening reflects the interaction between applied mechanical demands and the local anchorage capacity of bone [2]. Construct-level factors, including screw trajectory, diameter, length, cortical engagement, fusion length, and sagittal correction, determine the magnitude and direction of forces transmitted to the pedicle and vertebral body. However, the capacity to resist these forces is ultimately governed by the biological substrate into which fixation is achieved [22, 38, 47, 48]. Reduced trabecular density, altered cortical architecture, and impaired bone remodeling lower resistance to toggling and pull-out forces, rendering fixation progressively vulnerable even in the absence of overt technical error [1, 32, 61].

Accordingly, bone quality has emerged as a dominant substrate-level determinant of PSL in degenerative lumbar populations, where osteopenia and osteoporosis are common [3, 34, 57]. Preoperative bone health assessment is therefore central to fixation planning and risk stratification. In routine practice, however, bone assessment remains inconsistent [58]. Dual-energy X-ray absorptiometry (DEXA), while the reference standard for diagnosing osteoporosis, has well-recognized limitations in the degenerative spine [33, 66]. Osteophytes, endplate sclerosis, vertebral deformity, and vascular calcification can spuriously elevate lumbar T-scores, obscuring cancellous weakness in patients most likely to undergo instrumented fusion [9, 20, 66]. Quantitative computed tomography (CT) offers volumetric assessment and better isolation of trabecular bone but is resource-intensive and not consistently embedded in routine preoperative workflows [4].

These limitations have prompted increasing interest in opportunistic imaging-derived metrics that leverage routinely acquired preoperative CT and magnetic resonance imaging (MRI) to infer fixation-related bone competence without additional imaging burden. CT-based Hounsfield unit measurements offer a readily accessible surrogate of trabecular radiodensity and have been associated with screw loosening across multiple degenerative cohorts [53, 82, 83]. MRI-based vertebral bone quality (VBQ) scoring provides a radiation-free alternative, capturing trabecular deterioration indirectly through marrow fat infiltration on T1-weighted imaging [12, 39]. More recently, trajectory- and pedicle-specific metrics have been proposed, reflecting growing recognition that fixation failure initiates locally at the screw-bone interface rather than uniformly within the vertebral body [27, 49, 74].

Beyond bone, paraspinal muscle degeneration and sarcopenia may represent an additional axis of vulnerability [17, 21]. Reduced muscle cross-sectional area (CSA) and adverse muscle composition may impair dynamic stabilization and load sharing across instrumented segments, amplifying cyclic stresses at the screw-bone interface. Emerging clinical studies have linked diminished psoas or paraspinal muscle size to increased loosening risk, although reported associations remain heterogeneous and measurement protocols inconsistent [37].

Despite rapid expansion of this literature, the evidence base remains fragmented across imaging modalities, measurement protocols, loosening definitions, and surgical contexts. Individual studies frequently report statistically significant associations but provide limited insight into comparative diagnostic performance, threshold stability, or clinical interpretability. Consequently, it remains unclear which opportunistic imaging-derived metrics most effectively discriminate patients at increased risk for PSL and how such measures should be integrated into preoperative planning. Accordingly, we performed a systematic review and meta-analysis to evaluate and compare the predictive performance of CT- and MRI-derived bone quality metrics and paraspinal muscle measures for PSL after degenerative lumbar fusion. By synthesizing both continuous inter-group differences and diagnostic accuracy parameters, we sought to clarify the discriminative capacity, operating characteristics, and practical utility of these opportunistic imaging metrics as tools for preoperative risk stratification.

## Materials and methods

### Reporting standards and registration

This systematic review and meta-analysis was conducted in accordance with the Preferred Reporting Items for Systematic Reviews and Meta-Analyses of Diagnostic Test Accuracy Studies (PRISMA-DTA) guidelines for diagnostic accuracy reviews [51]. The protocol was registered a priori in the Prospective Register of Systematic Reviews (PROSPERO) (CRD420261287970) on 15 January 2026 before title/abstract screening, full-text review, data extraction, and statistical analysis, specifying eligibility criteria, outcomes, and planned analytical methods. PRISMA-DTA was selected as the primary framework because the core synthesis is an index-test-versus-reference-standard accuracy evaluation with diagnostic estimands (pooled sensitivity, specificity, and SROC-derived AUC). PRISMA 2020 paired with a QUIPS- or PROBAST-style prognostic appraisal was considered but not adopted as a second, partly redundant framework; the prognostic dimension (preoperative index tests, postoperatively ascertained endpoint) is instead handled through the risk-of-bias and construct-level confounding considerations below.

### Literature search strategy

A comprehensive electronic search of PubMed, Embase, and Cochrane Central Register of Controlled Trials (CENTRAL) was performed from database inception to 18 January 2026. Search strategies combined controlled vocabulary and free-text terms related to PSL, lumbar fusion, bone quality, and paraspinal muscle composition, including terms for Hounsfield units, VBQ, bone mineral density (BMD), sarcopenia, and muscle fat infiltration (Supplementary Table 1). Search strings were adapted for each database and prospectively restricted to human studies published in English at the database search stage as a prespecified feasibility constraint (non-English titles and abstracts were therefore not subsequently screened for eligibility), though this design choice is acknowledged as a potential source of language bias. Reference lists of included studies and relevant reviews were screened to identify additional eligible studies.

### Eligibility criteria

Studies were eligible if they included adult patients (≥ 18 years) undergoing instrumented lumbar or lumbosacral fusion with pedicle screw-based constructs, reported radiographic PSL as an outcome, evaluated at least one preoperative imaging-derived bone or muscle metric, and provided extractable quantitative data comparing patients with and without PSL, or data enabling reconstruction of diagnostic performance. All eligible cohorts were treated as a single prognostic setting (adult degenerative lumbar fusion with pedicle screw instrumentation). Subgroup analyses by construct type, fusion length, or lumbosacral involvement were not prespecified. By design, single-level TLIF/PLIF, multilevel fusion, adult degenerative scoliosis, L5–S1 constructs, and the few semi-rigid and dynamic-rod cohorts were combined in a single primary analysis. The review is therefore a pragmatic, substrate-level synthesis rather than a construct-specific prediction model: pooled estimates index the shared screw-bone substrate and do not capture construct-level stability differences, revisited as a portability constraint in the Discussion. Cement augmentation, cortical bone trajectory screws, navigation- or robotic-assisted placement, sacropelvic fixation, and anabolic or antiresorptive osteoporosis pharmacotherapy were sought during data extraction but were too inconsistently reported across the included cohorts to support a dedicated data column or formal subgroup analysis. Included constructs were predominantly rigid, and no included study employed sacropelvic or iliac fixation. We excluded case reports, case series with < 10 patients, conference abstracts, biomechanical or cadaveric studies, technical notes, and studies lacking sufficient data for quantitative synthesis.

### Study selection

Two reviewers independently screened titles, abstracts, and full texts. Disagreements were resolved by consensus discussion, with arbitration by a third reviewer when required. Inter-reviewer agreement for study inclusion was assessed using Cohen’s κ statistic, with substantial agreement between reviewers (κ = 0.82).

### Definition of pedicle screw loosening

PSL was defined radiographically according to individual study criteria, most commonly as a radiolucent halo ≥ 1 mm surrounding the screw on follow-up radiographs or CT, and/or progressive circumferential radiolucency or evidence of screw toggling on serial imaging. Where multiple definitions were reported, the primary or most stringent criterion used by the original authors was adopted. Reference standard modality (plain radiographic, CT, combined radiograph-and-CT, or MRI) and study-level definitions of peri-screw lucency were recorded. Each study's PSL definition, adjudication modality, surveillance timing, and diagnostic threshold are itemized in Table 1, with a descriptive radiograph-only versus CT comparison in the Results. Because CT detects peri-screw lucency more sensitively than plain radiography, this distinction is carried through interpretation rather than collapsed into a single pooled figure. A reference-standard hierarchy was not prespecified, and modality-stratified sensitivity analyses were not undertaken given that reference modality is non-randomly distributed across geography, surgical era, and the index test of interest. Reference-standard heterogeneity is therefore handled through transparent study-level reporting and interpretative caveats.

**Table 1:** Bone metrics

Author	Definition of PSL	PSL diagnostic tool	Definition of VBQ	VBQ (mean ± SD or (range))		Definition of HU	HU values (mean ± SD or (range))		Definition of osteoporosis T-Score
	Overall	Overall	Overall	PSL	Non-PSL	Overall	PSL	Non-PSL	Overall
Yen-Chun (2024)	Radiographic ≥ 1 mm radiolucent halo surrounding the pedicle screw	CT scan	NR	NR	NR	HU measurements as per local protocol	101.3 ± 49.4	127.5 ± 53.3	NR
Yongdi (2025)	Gap ≥ 1 mm surrounding the pedicle screw	X-ray and CT scan	VBQ scores were calculated by dividing the median signal intensity (SI) of the vertebral bodies at L1 − L4 by the mean SI of CSF at L3	4.0 ± 0.7	3.2 ± 0.8	NR	NR	NR	NR
Chen (2025)	Fissure ≥ 1 mm around the screws	CT scan	NR	NR	NR	NR	NR	NR	T score was determined with DXA
Zan (2024)	≥ 1 mm radiolucent zones around the screw	AP and lateral X-ray	NR	NR	NR	The HU value was measured in the central sagittal plane of the screw (rather than the vertebral body's sagittal plane)	109.8 ± 21.1	129.6 ± 49.1	NR
Yung-Hsueh (2025)	Radiolucent zone around the screw ≥ 1 mm	Plain radiograph	VBQ score = Median (SIL1 − L4)/SICSF	3.8 ± 0.6	2.9 ± 0.6	NR	NR	NR	NR
Pengyu (2025)	Radiolucent zone of ≥ 1 mm around the pedicle screw	X-ray	VBQ score = Median (SIL1 − L4)/SICSF	3.2 ± 0.3	2.8 ± 0.4	HU values were measured using preoperative axial CT images. An elliptical ROI was manually placed in the mid-vertebral body of each vertebra from L1 to L4, avoiding the cortical margin and the posterior venous plexus	94.6 ± 21.0	110.9 ± 23.3	NR
An-Jhih (2025)	Radiolucent zone > 1 mm or the presence of a “double halo” sign	AP and lateral stress X-ray, or lateral plain films	Mean signal intensity was calculated and normalized to the cerebrospinal fluid (CSF) ROI at the posterior aspect of the L3 vertebral body, yielding VBQ values	3.5 ± 0.7	2.9 ± 0.6	NR	NR	NR	Bone mineral density (BMD) was measured at the lumbar spine and left proximal femur using a DXA Horizon® W scanner
Wu (2024)	Clear zone of minimal thickness of ≥ 1 mm around the pedicle screw	X-ray	VBQ score = median (SIL1 − L4)/SICSF	3.5 ± 0.7	2.9 ± 0.6	Hounsfield units (HU) value was measured on CT scans	98.8 ± 26.7	118.7 ± 39.9	NR
Guozheng (2024)	Radiolucent zone ≥ 1 mm around the pedicle screw	CT scan	VBQ score = median (SIL1-4)/SICSF	3.6 ± 0.5	2.8 ± 0.3	ROI were delineated within the trabecular bone, avoiding cortical bone, areas of local bone hyperplasia, and the vertebral venous plexus. The HU value within the ROI was then obtained	94.4 ± 29.0	129.3 ± 37.0	NR
Jingchi (2022)[L4]	≥ 1 mm width radiolucent zones around the screw	CT scan	NR	NR	NR	The ROI was placed in four planes: the midsagittal plane, central transverse plane, transverse plane close to the superior plane, and inferior endplate separately. The values were then averaged (CT scan)	NR	NR	NR
Jingchi (2022) [L5]	≥ 1 mm width radiolucent zones around the screw	CT scan	NR	NR	NR	The ROI was placed in four planes: the midsagittal plane, central transverse plane, transverse plane close to the superior plane, and inferior endplate separately. The values were then averaged (CT scan)	NR	NR	NR
Zan (2023)	Radiolucent zone with a width > 1 mm around the screw and a double halo sign	X-ray	(VBQ score = SI of L1-L4 ∕ SI of CSF	3.1 ± 0.5	2.8 ± 0.4	NR	NR	NR	All patients underwent DXA (Lunar DPX Prodigy, GE) of the lumbar spine and hip to evaluate the lumbar spine and lowest BMDs and T-score
He (2023)	Radiolucent area (≥ 1 mm in this study) around any screw	X-ray and CT scans	NR	NR	NR	L5 vertebrae were selected for HUs measurement in every patient. An axial image at the mid-height level of the L5 vertebra was chosen for the vertebral body measurement	149.1 ± 56.4	181.8 ± 61.9	DXA examination (Hologic Discovery model A densitometers, Hologic, Inc., Bedford, MA, USA) before the surgery was conducted with standard flow including lumbar spine (L1 to L4 vertebrae), BMD values were recorded as T-scores
Kyeong (2022)	Radiolucent area (thicker than 1 mm) around screw or the “double halo”	X-ray scan	NR	NR	NR	HU values were measured in preoperative 3D-CT	84.9 ± 34.5	125.6 ± 51.4	Bone mineral density and T-score were measured by DXA scan
Maximilian (2021)	NR	CT scan	NR	NR	NR	NR	NR	NR	NR
Fei (2020)	Clear zone of minimal thickness of ≥ 1 mm around the pedicle screw	X-ray	NR	NR	NR	The average of the ROI HU value was calculated using a PACS. An axial plane at the mid–vertebral body was chosen for the measurement of vertebral body HU	98.6 ± 25.8	121.4 ± 39.7	NR
Guo (2020)	Radiolucent line of ≥ 1 mm around the screw	X-ray and CT scan	NR	NR	NR	NR	NR	NR	NR
Leng (2020) [≥ 6 lvls]	≥ 1 mm circumferential radiolucent line around the pedicle screw	X-ray and CT scan	NR	NR	NR	NR	NR	NR	NR
Leng (2020) [4–5 lvls]	≥ 1 mm circumferential radiolucent line around the pedicle screw	X-ray and CT scan	NR	NR	NR	NR	NR	NR	NR
Cho (2018)	≥ 1 mm circumferential radiolucent line	CT scan	NR	NR	NR	NR	NR	NR	NR
Sakai (2018)	Radiolucent area (≥ 1 mm in circumference) around the screw	CT scan	NR	NR	NR	On each screw axis–oriented slice, a rectangular ROI matching the screw length and diameter was placed over the planned pedicle screw path, and the mean HU within this rectangle was recorded (CT scan)	147.0 ± 94.0	208.0 ± 91.0	NR
Matsukawa (2017)	Radiolucent zone ≥ 1 mm around the screw	CT scan	NR	NR	NR	NR	NR	NR	NR
Li (2024)	Clear zone around pedicle screws ≥ 1 mm	X-ray	An ROI is placed in the trabecular bone of S1 on the mid-sagittal slice. A second ROI is placed in the CSF at L3 (or nearby level) to get CSF signal intensity S1 VBQ score = SI of S1 trabecular bone ÷ SI of CSF at L3. (T1 MRI)	3.3 ± 0.4	3.0 ± 0.3	NR	NR	NR	DXA
Shu 2023	≥ 1 mm of clear zone around screw	X-ray	NR	NR	NR	Hounsfield units were measured on preoperative CT scans by placing a ROI inside the vertebral body cortex at the pedicle level on axial images, then using the PACS system to automatically calculate the mean HU value for that ROI	122.4 ± 32.8	142.4 ± 38.2	NR
Li 2023	Clear zone ≥ 1 mm around the pedicle screw	X-ray	VBQ was measured on non-contrast T1-weighted lumbar MRI by placing ROIs in the medullary portions of L1–L4 and in the CSF space at L3; the VBQ score was calculated as the median vertebral SI divided by the CSF SI, using parasagittal slices or alternative vertebral/CSF levels (L2) when mid-sagittal vertebrae or the L3 CSF space were abnormal or blocked	3.3 ± 0.6	2.8 ± 0.5	The mean HU value was calculated as the average of the individual L1–L4 HU measurements	111.9 ± 37.0	146.9 ± 50.0	NR
Li 2024	Translucent halo ≥ 1 mm thickness encircling the pedicle screw	T1-weighted sagittal MRI	The PBQ (pedicle bone quality) score is an MRI-based surrogate for VBQ. On T1-weighted sagittal MRI, regions of interest (ROIs) are: Pedicle ROIs: drawn 1 mm inside the pedicle cortex from L1–L4, avoiding focal lesions CSF ROI: placed in the posterior CSF space at L3 The PBQ score = median SI of L1–L4 pedicles ÷ SI of CSF at L3	3.0 ± 0.5	3.5 ± 0.5	Vertebral body HU was measured by drawing as large an elliptical ROI as possible within the cancellous bone on CT axis	113.5 ± 29.0	135.0 ± 25.4	Lumbar T score based on DXA
Şakir Ekşi 2025	Haloing ≥ 1-mm circumferential radiolucent line around the pedicle screw	X-ray	NR	NR	NR	NR	NR	NR	NR
Zhou 2024	Halo sign with a circumferential radiolucent line of ≥ 1 mm around the S1 pedicle screw	X-rays or CT	NR	NR	NR	CT scan	140.2 ± 43.1	157.4 ± 45.6	NR
Yuya 2023	Screws having ≥ 1 mm radiolucent clear zone over the whole length	CT scan	NR	NR	NR	HU values were measured from CT scans	98.0 ± 31.0	128.0 ± 47.0	NR
Wei (2021)	≥ 1-mm circumferential radiolucent line around the pedicle screw	X-ray	NR	NR	NR	NR	NR	NR	NR
Jinxiang (2024)	Lucent zone of ≥ 1 mm around the screws, the double halo sign, or both	X-ray or CT imaging	The calculation of the PBQ score involves dividing the median SI of the pedicles from L1 to L4 by the SI of the CSF at the L3 pedicle. The final PBQ score reflects the average bone quality index derived from the two pedicles	PBQ: 3.6 (3.4–3.8)	PBQ: 3.01 (2.7–3.4)	Lumbar CT	94.7 (77.9–120.8)	121.6 (86.2–154.4)	NR

Author	Definition of osteoporosis T-Score	Osteoporosis T-score (mean ± SD)		Definition of CT-BMD (mg/cm3)	CT-BMD, in mg/cm3 (mean ± SD or (range))		AUC (95% CI)	Cutoff	Follow-up to PSL assessment (months)
	Overall	PSL	Non-PSL	Overall	PSL	Non-PSL	Overall	Overall	
Yen-Chun (2024)	NR	NR	NR	NR	NR	NR	HU: 0.67	NR	24.0
Yongdi (2025)	NR	NR	NR	NR	101.2 ± 22.4	136.6 ± 29.3	VBQ: 0.79 (0.70–0.88); vBMD: 0.84 (0.77–0.91)	VBQ: 3.59; vBMD: 119.00	32.9 ± 10.6
Chen (2025)	T score was determined with DXA	− 1.3 ± 1.3	− 1.2 ± 1.3	For QCT, L5 vertebrae were selected for the vBMD measurement in every patient	91.0 (61.0–118.0)	109.0 (92.0–136.0)	vBMD: 0.68 (0.60–0.76)	vBMD: 90.05	12.0
Zan (2024)	NR	NR	NR	NR	NR	NR	Left HU: 0.58; right HU: 0.49	Left HU: 116.50; Right HU: 121.50	12.6 (11.4–14.2)
Yung-Hsueh (2025)	NR	NR	NR	NR	NR	NR	VBQ: 0.79	VBQ: 3.10	34.7 ± 16.4
Pengyu (2025)	NR	NR	NR	NR	NR	NR	VBQ: 0.81 (0.73–0.89)	VBQ: 3.00	12.0
An-Jhih (2025)	Bone mineral density (BMD) was measured at the lumbar spine and left proximal femur using a DXA Horizon® W scanner	−2.1 ± 1.5	−2.2 ± 1.1	NR	NR	NR	VBQ: 0.72 (0.66–0.78)	VBQ: 3.40	12.0
Wu (2024)	NR	NR	NR	NR	NR	NR	VBQ: 0.75 (0.66–0.84); HU: 0.65 (0.56–0.75)	NR	12.0
Guozheng (2024)	NR	NR	NR	NR	NR	NR	VBQ: 0.82; HU: 0.80	VBQ: 3.15	37.5 ± 11.4
Jingchi (2022)[L4]	NR	NR	NR	NR	NR	NR	HU: 0.73 (0.48–0.88)	HU: 105.56	12.2 (11.0–13.5)
Jingchi (2022) [L5]	NR	NR	NR	NR	NR	NR	HU: 0.83 (0.56–0.93)	HU: 107.3	12.2 (11.0–13.5)
Zan (2023)	All patients underwent DXA (Lunar DPX Prodigy, GE) of the lumbar spine and hip to evaluate the lumbar spine and lowest BMDs and T-score	−1.0 ± 1.4	−0.9 ± 1.4	NR	NR	NR	VBQ: 0.74 (0.65–0.77)	VBQ: 2.87	12.2 ± 1.3
He (2023)	DXA examination (Hologic Discovery model A densitometers, Hologic, Inc., Bedford, MA, USA) before the surgery was conducted with standard flow including lumbar spine (L1 to L4 vertebrae), BMD values were recorded as T-scores	−1.3 ± 1.8	−1.3 ± 1.3	NR	NR	NR	HU: 0.67 (0.55–0.79); T-score: 0.53 (0.40–0.65)	HU: 160.00	6.0
Kyeong (2022)	Bone mineral density and T-score were measured by DXA scan	−0.9 ± 2.0	−0.2 ± 1.7	NR	NR	NR	HU: 0.77 (0.63–0.86)	HU: 104.91	6.0
Maximilian (2021)	NR	NR	NR	CT scans were used for opportunistic BMD screening	86.5 ± 29.5	118.2 ± 32.9	vBMD: 0.77 (0.63–0.91)	NR	12.0
Fei (2020)	NR	NR	NR	NR	NR	NR	HU: 0.674 (0.59–0.75)	HU: 130.00	12.0
Guo (2020)	NR	−3.1 ± 0.6	−2.9 ± 0.6	NR	NR	NR	NR	NR	NR
Leng (2020) [≥ 6 lvls]	NR	−0.5 ± 2.2	−0.9 ± 1.8	NR	NR	NR	NR	NR	12.0
Leng (2020) [4–5 lvls]	NR	−1.6 ± 1.5	−1.0 ± 1.6	NR	NR	NR	NR	NR	12.0
Cho (2018)	NR	−1.6 ± 1.4	−0.7 ± 1.6	NR	NR	NR	NR	NR	12.0
Sakai (2018)	NR	NR	NR	NR	NR	NR	Male HU: 0.64 (0.45–0.82); female HU: 0.88 (0.80–0.96)	Female HU: 153.50	12.0
Matsukawa (2017)	NR	NR	NR	NR	NR	NR	NR	NR	25.6 ± 10.2
Li (2024)	DXA	−1.3 ± 1.2	−1.1 ± 1.4	NR	NR	NR	VBQ: 0.75 (0.64–0.85)	VBQ: 3.18	12.0
Shu 2023	NR	NR	NR	NR	NR	NR	HU: 0.69 (0.61–0.77)	HU: 124.00	19.4 ± 10.8
Li 2023	NR	NR	NR	NR	NR	NR	VBQ: 0.72	NR	12.0
Li 2024	Lumbar T score based on DXA	−1.6 ± 1.2	−1.4 ± 1.1	NR	NR	NR	HU: 0.70 (0.61–0.79)	HU: 151.50	NR
Şakir Ekşi 2025	NR	NR	NR	NR	NR	NR	NR	NR	35.9 ± 25.3
Zhou 2024	NR	NR	NR	NR	NR	NR	NR	NR	27.5 ± 9.5
Yuya 2023	NR	NR	NR	NR	NR	NR	HU: 0.67 (0.55–0.80); BMD: 0.67 (0.54–0.80)	NR	6.0
Wei (2021)	NR	−0.8 ± 1.5	−1.1 ± 1.6	NR	NR	NR	NR	NR	38.4 ± 16.0
Jinxiang (2024)	NR	NR	NR	NR	NR	NR	PBQ: 0.79 (0.73–0.84)	PBQ: 3.20	12.0

Summary of bone metrics and study-level definitions. For each included study, the table reports the radiographic PSL definition, the adjudication modality (SL diagnostic tool), the imaging-metric definitions and regions of interest, the study-level Youden-derived cutoff, and the follow-up duration to PSL assessment (months). *PSL* pedicle screw loosening, *SD* standard deviation, *CSF* cerebrospinal fluid, *VBQ* vertebral bone quality, *SI* signal intensity, *CT* computed tomography, *QCT* quantitative computed tomography, *CI* confidence interval, *HU* Hounsfield units, *vBMD* quantitative CT–derived volumetric trabecular bone mineral density, *BMD* bone mineral density, *NR* not reported, *ROI* region of interest, *DEXA* dual-energy X-ray absorptiometry

### Outcomes of interest

Primary outcomes were preoperative imaging-derived metrics hypothesized to reflect substrate-level vulnerability at the screw-bone interface, including CT-based mean Hounsfield units. Three biomechanically distinct Hounsfield unit subfamilies were considered a priori: vertebral cancellous (region of interest (ROI) placed within the trabecular bone of the vertebral body, single-level or averaged across L1–4), trajectory-based (ROI aligned along the planned or actual screw axis), and pedicle-focused (ROI within the pedicle or juxtapedicular corridor on axial imaging). These subfamilies are ordered by proximity to the interface (vertebral cancellous as a global surrogate, trajectory-based approximating the screw path, pedicle-focused most local), with trajectory- and pedicle-density measures reported to outperform global vertebral values for loosening [5, 40]. Per-subfamily study counts are reported in the Results, and pooled values are not interpreted as trajectory-specific. Formal Hounsfield unit subfamily pooling and moderator analyses were planned only if each subfamily was represented by a sufficient number of studies to support stable estimation. MRI-based VBQ scores encompassed three protocol variants pooled under a unified heading: (i) standard lumbar VBQ (median L1–4 T1 signal intensity divided by cerebrospinal fluid (CSF), at L3); (ii) sacral VBQ (S1-based ROI); (iii) pedicle bone quality (PBQ) score (median L1–L4 pedicle signal divided by CSF at L3). These variants are treated as related but non-equivalent measures, not a single test: the pooled estimate is accompanied by a variant-level breakdown in the Results. A variant-stratified pool was not undertaken because the standard L1–L4, S1, and PBQ subsets are each too small for stable separate estimation. CT-derived bone mineral density (CT-BMD) (mg/cm^3^) was extracted where calibrated or phantomless techniques were used. Secondary outcomes primarily included imaging-derived measures of paraspinal muscle quantity and composition, assessed in muscle groups relevant to lumbar load sharing and stabilization (including the psoas, erector spinae, and multifidus). Extracted metrics included relative total cross-sectional area (rtCSA) (defined as the ratio of total muscle CSA to disc area at the same vertebral level), relative functional cross-sectional area (rfCSA) (defined as the ratio of pure muscle CSA (excluding adipose tissue) to disc at the same vertebral level), muscle fat index (MFI) (defined as the ratio of T2 signal intensity of muscle to subcutaneous fat at the same vertebral level), and Goutallier grading, as well as DEXA T-scores, fusion status, and clinical outcomes including pre- and postoperative Oswestry Disability Index (ODI) and visual analogue scale (VAS) back pain scores, where reported. Pooling was structured a priori at the muscle × metric level (psoas, erector spinae, and multifidus separately for rtCSA, rfCSA, and MFI), with no pooling across muscles or across metric types. We did not impose further stratification by normalization scheme or vertebral level of measurement, and these are not biomechanically interchangeable: rtCSA and rfCSA were variably normalized to intervertebral disc area or to vertebral body area, and multifidus measurements in particular are segment-specific given the level-dependent pattern of multifidus degeneration. Given the small number of studies contributing to each muscle × metric cell, formal stratified pooling by normalization scheme or measurement level was not undertaken, and the muscle synthesis is framed as exploratory throughout. Table 2 specifies, per study, the muscle, side, level, normalization denominator, segmentation method, and bilateral aggregation, so that psoas CSA at L3 is not conflated with multifidus at the caudal instrumented level. Accordingly, reduced psoas CSA is presented as a hypothesis-generating frailty marker, unadjusted for concurrent bone quality or construct variables.

**Table 2:** Sarcopenia metrics

Author (Year)	Parameter definition	Normalization method	Lumbar levels assessed	Psoas muscle		Multifidus muscle		Erector spinae muscle		Side	Segmentation/composition method	Aggregation (levels and sides)
				PSL	Non-PSL	PSL	Non-PSL	PSL	Non-PSL			
Yen-Chun (2025)	PLVI: measuring the CSA of the bilateral psoas muscles at the L4 level, and the value was normalized by dividing it by the CSA of the L4 vertebral body	Vertebral body normalized	L4	PLVI: 1.09 ± 0.39	PLVI: 1.59 ± 0.47	NR	NR	NR	NR	Bilateral	Psoas CSA (area); method NR	Single level (L4)
Leng (2020)	rfCSA: ratio of functional (lean) muscle cross-sectional area to intervertebral disc cross-sectional area at the same level, measured on axial T2-weighted MRI at L4–5 and L5–S1	Disc-area normalized	L4-S1	rfCSA: 0.90 ± 0.23	rfCSA: 0.99 ± 0.23	rfCSA: 0.56 ± 0.17	rfCSA: 0.60 ± 0.19	rfCSA: 0.68 ± 0.18	rfCSA: 0.83 ± 0.24	NR	Threshold-based (functional/lean CSA)	Per level (L4–5, L5–S1)
Leng (2020)	rfCSA: ratio of functional (lean) muscle cross-sectional area to intervertebral disc cross-sectional area at the same level, measured on axial T2-weighted MRI at L4–5 and L5–S1	Disc-area normalized	L4-S1	rfCSA: 0.92 ± 0.21	rfCSA: 0.96 ± 0.23	rfCSA: 0.61 ± 0.16	rfCSA: 0.64 ± 0.17	rfCSA: 0.82 ± 0.28	rfCSA: 0.73 ± 0.26	NR	Threshold-based (functional/lean CSA)	Per level (L4–5, L5–S1)
Leng (2020) [≥ 6 lvls]	MFI: ratio of mean T2-weighted signal intensity of muscle to that of subcutaneous fat at the same level, with higher values indicating greater fatty infiltration	Signal-intensity ratio	L4-S1	MFI: 0.16 ± 0.06	MFI: 0.14 ± 0.04	MFI: 0.42 ± 0.13	MFI: 0.45 ± 0.16	MFI: 0.41 ± 0.09	MFI: 0.41 ± 0.14	NR	Signal-intensity ratio (muscle:subcutaneous fat)	Per level (L4–S1)
Leng (2020) [4–5 lvls]	MFI: ratio of mean T2-weighted signal intensity of muscle to that of subcutaneous fat at the same level, with higher values indicating greater fatty infiltration	Signal-intensity ratio (Fatty infiltration/composition metrics)	L4-S1	MFI: 0.17 ± 0.04	MFI: 0.15 ± 0.05	MFI: 0.40 ± 0.13	MFI: 0.43 ± 0.13	MFI: 0.40 ± 0.09	MFI: 0.41 ± 0.10	NR	Signal-intensity ratio (muscle:subcutaneous fat)	Per level (L4–S1)
Şakir Ekşi (2025)	One-level score of a paraspinal muscle was calculated as the sum of Goutallier score for the right- and left-sides of that muscle for the corresponding lumbar level	Ordinal fatty degeneration score	L1-S1	Goutallier score: L1- 2: 1.05 ± 0.99; L2–3: 0.88 ± 1.18; L3–4: 1.27 ± 1.27; L4–5: 1.83 ± 1.20; L5–S1: 1.61 ± 0.84	Goutallier score: L1- 2: 0.81 ± 1.06; L2–3: 1.08 ± 1.25; L3–4: 1.60 ± 1.23; L4–5: 2.00 ± 1.30; L5–S1: 1.84 ± 1.16	Goutallier score: L1- 2: 3.38 ± 1.94; L2–3: 3.66 ± 1.84; L3–4: 3.94 ± 1.55; L4–5: 4.77 ± 1.51; L5–S1: 5.16 ± 1.42	Goutallier score: L1- 2: 2.41 ± 1.49; L2–3: 2.57 ± 1.36; L3–4: 3.13 ± 1.54; L4–5: 4.20 ± 1.60; L5–S1: 5.05 ± 1.64	Goutallier score: L1- 2: 3.33 ± 1.97; L2–3: 3.22 ± 1.66; L3–4: 3.05 ± 1.73; L4–5: 3.38 ± 1.33; L5–S1: 4.88 ± 2.21	Goutallier score: L1- 2: 2.41 ± 1.34; L2–3: 2.40 ± 1.27; L3–4: 2.76 ± 1.32; L4–5: 3.88 ± 1.81; L5–S1: 5.21 ± 1.88	Bilateral	Qualitative Goutallier grading	Per level (L1–S1); right + left summed
Zhou (2024)	rtCSA: (tCSA) ÷ disc area at the same level	Disc-area normalized	L4-5	rtCSA: L2–3: 1.18 ± 0.37; L3–4: 1.13 ± 0.32; L4–5: 1.12 ± 0.24; L5–S1: 1.10 ± 0.26	rtCSA: L2–3: 1.15 ± 0.28; L3–4: 1.18 ± 0.29; L4–5: 1.18 ± 0.21; L5–S1: 1.29 ± 1.11;	rtCSA: L4-5: 0.48 ± 0.14	rtCSA: L4-5: 0.46 ± 0.19	rtCSA: L4-5: 0.87 ± 0.33	rtCSA: L4-5: 0.79 ± 0.28	NR	Total CSA (area)	Per level
Zhou (2024)	rfCSA: fCSA (lean muscle) ÷ disc area	Disc-area normalized	L2-S1	rfCSA: L2–3: 1.16 ± 0.34; L3–4: 1.14 ± 0.36; L4–5: 1.14 ± 0.23; L5–S1: 1.13 ± 0.26	rfCSA: L2–3: 1.18 ± 0.29; L3–4: 1.21 ± 0.29; L4–5: 1.23 ± 0.22; L5–S1: 1.53 ± 2.86	rfCSA: L4-5: 0.34 ± 0.10	rfCSA: L4-5: 0.32 ± 0.14	rfCSA: L4-5: 0.67 ± 0.27	rfCSA: L4-5: 0.59 ± 0.24	NR	Threshold-based (functional/lean CSA)	Per level
Zhou (2024)	MFI: ratio of T2 signal intensity of muscle to that of subcutaneous fat at the same level	Signal-intensity ratio (Fatty infiltration/composition metrics)	L2-S1	MFI: L2–3: 1.22 ± 0.42; L3–4: 1.17 ± 0.39; L4–5: 1.22 ± 0.51; L5–S1: 1.26 ± 0.59	MFI: L2–3: 1.10 ± 0.39; L3–4: 1.13 ± 0.46; L4–5: 1.13 ± 0.46; L5–S1: 1.15 ± 0.48	MFI: L4-5: 0.28 ± 0.11	MFI: L4-5: 0.31 ± 0.11	MFI: L4-5: 0.23 ± 0.08	MFI: L4-5: 0.25 ± 0.10	NR	Signal-intensity ratio (muscle:subcutaneous fat)	Per level
Wei (2021)	rtCSA: the ratio of total muscle cross-sectional area to vertebral cross-sectional area was used as rtCSA	Vertebral body normalized	L3-5	rtCSA: L3 0.80 ± 0.22; L4 1.09 ± 0.29; L5 1.31 ± 0.37	rtCSA: L3 0.82 ± 0.24; L4 1.11 ± 0.29; L5 1.43 ± 0.34	rtCSA: L3 0.41 ± 0.18; L4 0.55 ± 0.24; L5 0.62 ± 0.28	rtCSA: L3 0.41 ± 0.20; L4 0.57 ± 0.24; L5 0.64 ± 0.29	rtCSA: L3 2.23 ± 0.58; L4 1.99 ± 0.50; L5 1.30 ± 0.47	rtCSA: L3 2.42 ± 0.58; L4 2.07 ± 0.45; L5 1.64 ± 0.57	NR	Total CSA (area)	L3–5
Wei (2021)	rfCSA: the ratio of functional muscle cross-sectional area to vertebral cross-sectional area was used as rfCSA	Vertebral body normalized	L3-5	rfCSA: L3 0.86 ± 0.30; L4 1.19 ± 0.36; L5 1.32 ± 0.92	rfCSA: L3 0.90 ± 0.24; L4 1.21 ± 0.34; L5 1.36 ± 0.37	rfCSA: L3 0.41 ± 0.17; L4 0.55 ± 0.24; L5 0.62 ± 0.28	rfCSA: L3 0.41 ± 0.20; L4 0.57 ± 0.24; L5 0.64 ± 0.29	rfCSA: L3 1.36 ± 0.48; L4 1.10 ± 0.40; L5 0.58 ± 0.28	rfCSA: L3 1.48 ± 0.56; L4 1.15 ± 0.39; L5 0.73 ± 0.35	NR	Threshold-based (functional CSA)	L3–5

Summary of sarcopenia metrics and study-level definitions. *PSL* pedicle screw loosening, *CSA* cross-sectional area, *rfCSA* relative functional cross-sectional area, *rtCSA* relative total muscle cross-sectional area, *MFI* muscle fat index, *PLVI* psoas lumbar vertebral index, *NR* not reported. For each included study, the table specifies the parameter definition, normalization denominator, lumbar level(s) assessed, measurement side, segmentation or composition method, and aggregation across levels and sides; fatty-infiltration metrics are characterized within the normalization and segmentation columns (signal-intensity-ratio MFI or qualitative Goutallier grading)

### Data extraction

Data were extracted independently by two reviewers using a prespecified form. Extracted variables included study characteristics, patient demographics, surgical technique, definition and incidence of screw loosening, imaging modality and measurement protocols, reported cutoff values, and follow-up duration. For diagnostic accuracy analyses, true positives (TP), false positives (FP), false negatives (FN), and true negatives (TN) were extracted directly or reconstructed from reported data. In studies reporting outcomes stratified by vertebral level or by number of levels fused, data from each stratum was extracted separately and entered as separate observations in the random-effects models, consistent with Cochrane Handbook guidance for handling studies with multiple groups. Non-overlapping patient strata were treated as independent observations; overlapping level-specific strata were retained only when original data could not be collapsed because within-study correlations were unavailable, and this limitation was considered when interpreting precision. Of the 29 studies, only one cohort (Jingchi 2022) contributed overlapping within-patient strata (L4 and L5 from the same patients); other clustered structures, including the fusion-length subgroups of Leng 2020, were non-overlapping between-patient subgroups satisfying independence. With a single clustered cohort, a three-level model or robust variance estimation would yield unstable variance components for negligible gain; its influence was instead tested by leave-one-out analysis, which left the direction and significance of pooled estimates unchanged. Residual non-independence therefore affects precision, not direction.

### Risk of bias and certainty of evidence

Risk of bias was assessed using the Quality Assessment of Diagnostic Accuracy Studies 2 (QUADAS-2) tool [71], evaluating patient selection, index test conduct, reference standards, and flow or timing. QUADAS-2 was selected given that the principal analytic objective was diagnostic accuracy. The Quality in Prognosis Studies (QUIPS) tool was considered but not applied given its emphasis on long-term outcome prediction and confounder adjustment is less aligned with the cross-sectional, threshold-based structure of the included studies – dual application of overlapping instruments risks reduced interpretive clarity. PROBAST was likewise considered but not adopted as the primary instrument, because the included studies report single-marker threshold accuracy rather than multivariable prediction models. QUADAS-2 does not formally capture whether individual studies adjusted for construct- and patient-level confounders of loosening (fusion length, construct type, cement augmentation, osteoporosis pharmacotherapy, smoking, diabetes, sagittal correction, pseudarthrosis); because these were inconsistently reported, this residual confounding is incorporated qualitatively into the risk-of-bias interpretation and Limitations rather than formally scored. Certainty of evidence for each primary diagnostic metric was evaluated using the GRADE framework [60], with downgrading based on risk of bias, inconsistency, indirectness, imprecision, and publication bias.

### Statistical analysis

Analyses were designed to address two complementary objectives. First, whether imaging-derived metrics differ systematically between patients with and without PSL, and second, the extent to which these metrics discriminate patients at increased risk of loosening. All analyses were performed using *R* version 4.3.2 [55] with the *metafor* [67] and *mada* [11] packages. A two-sided α < 0.05 was considered statistically significant.

Continuous outcomes were synthesized using mean differences (MDs) or standardized mean differences (SMDs) with 95% confidence intervals (CIs) using a random-effects inverse-variance model with Hartung-Knapp adjustment, with the between-study variance (τ^2^) estimated by restricted maximum likelihood (REML). Statistical heterogeneity was assessed using the χ^2^ test and quantified with the I^2^ statistic. For pairwise outcomes demonstrating substantial heterogeneity, Baujat plots were generated to identify studies disproportionately influencing pooled estimates or heterogeneity. For diagnostic accuracy analyses, pooled sensitivity and specificity were estimated using random-effects bivariate (Reitsma) generalized linear mixed models. Summary diagnostic indices, including positive likelihood ratios (PLRs), negative likelihood ratios (NLRs), and diagnostic odds ratios (dORs) were derived accordingly. Overall discriminative performance was summarized using summary receiver operating characteristic (SROC) curves, with pooled area under the curve (AUC) reported.

Diagnostic 2 × 2 synthesis was implemented using logit-transformed sensitivity and specificity in a bivariate random-effects GLMM. One author-defined threshold per study and metric was entered to avoid duplication; where multiple thresholds were reported, the threshold designated as optimal by the original authors, typically Youden-derived, was extracted. Sparse diagnostic tables were inspected before pooling; where zero cells precluded calculation of derived likelihood ratios or diagnostic odds ratios, a 0.5 continuity correction was applied for those derived indices. The bivariate GLMM was fitted on observed, uncorrected cell counts; the 0.5 continuity correction was applied only to the downstream likelihood ratios and diagnostic odds ratios, not to the model likelihood. No diagnostic study with reconstructable 2 × 2 data was excluded solely because of sparse cells, and no bivariate model failed to converge. Convergence was confirmed by maximum-likelihood optimization with a positive-definite Hessian and finite standard errors for every model. Leave-one-out refitting of each bivariate model did not materially alter the pooled operating points or SROC-derived AUCs.

Where available, optimal thresholds defined by the Youden index were extracted at the study-level for each metric family (Hounsfield units, VBQ, CT-BMD) and presented descriptively at the study-level. Formal threshold pooling was not undertaken, as no validated meta-analytic framework currently exists for combining Youden-derived cutoffs across studies with heterogeneous measurement protocols, scanner parameters, and reference standards. All reported cutoffs are therefore internally derived study-level values, not externally validated thresholds. Because several studies derived Youden-optimal cutoffs and tested them in the same cohort, these values carry in-sample optimism bias and are descriptive only, consistent with reviews showing HU and VBQ thresholds vary with scanner, ROI, field strength, and protocol [16, 18, 63, 70]. Threshold effects were evaluated using Spearman correlation between sensitivity and FP rate. To aid clinical interpretability, Fagan nomograms were constructed assuming a pre-test probability of 25.0%, reflecting the observed pooled event rate across included cohorts (1194/4711, 25.4%). Meta-regression analyses were performed to explore potential sources of inter-study heterogeneity including imaging modality, metric type, and study-level characteristics where data permitted. Due to limited standardisation and incomplete reporting, variables such as follow-up duration, fusion length, and surgical approach were not included to avoid overfitting and unstable estimates. Meta-regression was considered exploratory and hypothesis-generating because available study counts and construct-level reporting were insufficient for stable multivariable moderator modeling. In this metric-type model the unit of analysis is the metric-level observation, not the study: each study contributes one observation per bone-metric family reported (HU, VBQ, or CT-BMD), so multi-family studies contribute more than once. Study-level clustering is therefore not fully accounted for, standard errors are potentially anti-conservative, and the result is exploratory only. Publication bias and small-study effects were assessed using Deeks’ funnel plot asymmetry test.

## Results

### Study selection and study characteristics

**Fig. 1:**
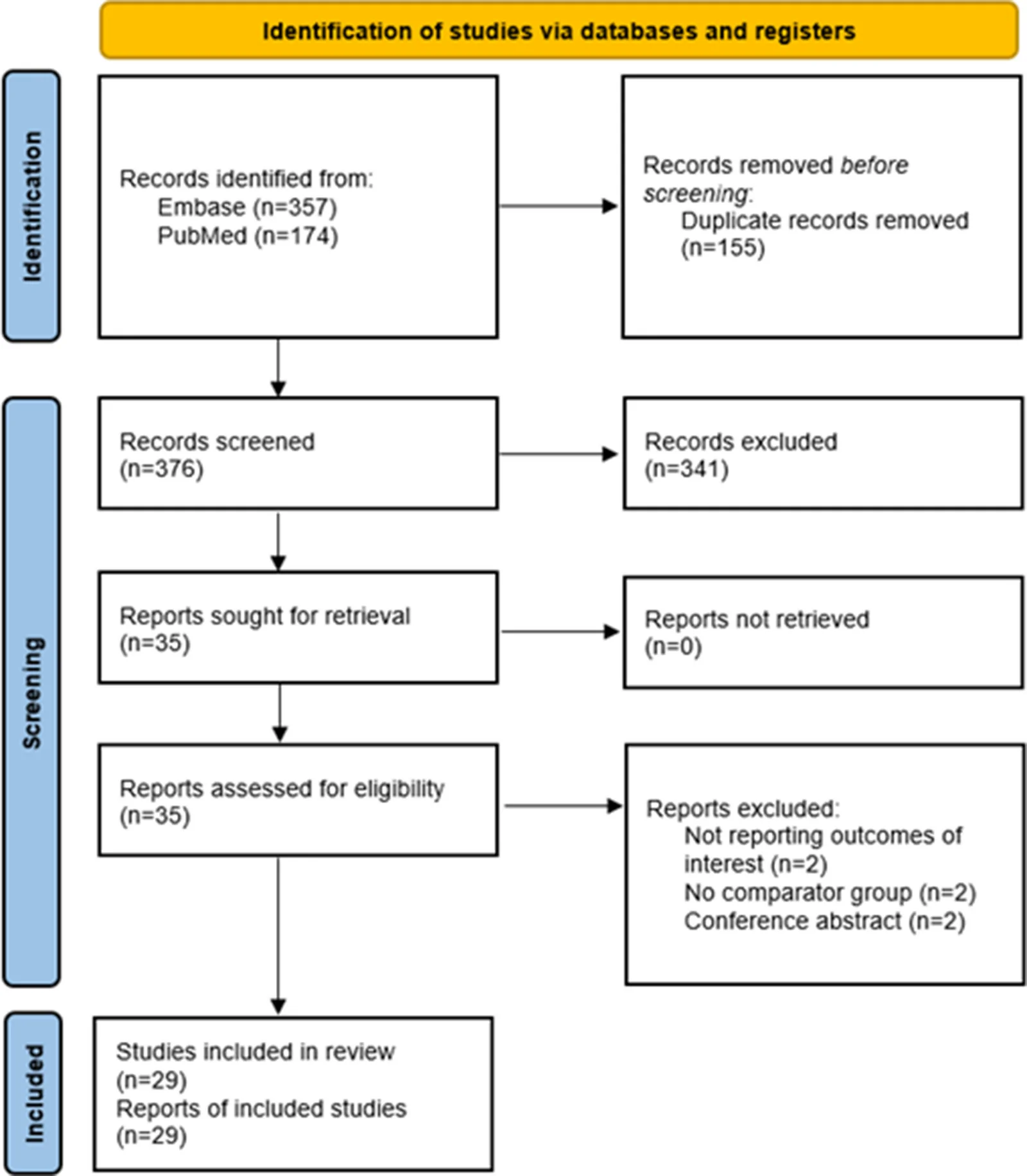
PRISMA flow diagram of study selection. Preferred Reporting Items for Systematic Reviews and Meta-Analyses (PRISMA) flow diagram outlining the study selection process. The number of records identified, screened, assessed for eligibility, and included in the final analysis are detailed at each stage

A total of 531 records were identified through database searching. After screening and full-text review, 29 studies [5, 6, 8, 13, 19, 27–30, 35, 37, 39–44, 50, 56, 62, 65, 68, 69, 74–76, 78, 79, 81] met inclusion criteria, comprising 4711 patients, including 1194 with PSL and 3517 without (Fig. 1). Individual study definitions of radiographic PSL (peri-screw lucency criteria and imaging modality) and imaging-derived bone metrics are provided in Table 1, and muscle metric definitions are provided in Table 2. Across analyses, the number of contributing studies differs between continuous and diagnostic syntheses for a given metric: continuous between-group comparisons included any study reporting group-level means, whereas diagnostic-accuracy pooling was restricted to studies from which a complete 2 × 2 contingency table could be reconstructed.

Study sample sizes ranged from 46 to 394 participants. Mean ages ranged from 55.6 to 73.0 years in PSL cohorts, and 52.7 to 71.9 years in non-PSL cohorts. The proportion of female patients ranged from 33.3% to 90.0% in PSL groups and 23.3 to 90.6% in non-PSL groups. Mean follow-up duration was comparable between groups (17.2 months in both PSL and non-PSL cohorts). The most frequently reported fusion technique was transforaminal lumbar interbody fusion (TLIF) (34.3%) followed by posterior lumbar interbody fusion (PLIF) (31.8%). Constructs were predominantly rigid, with semi-rigid [28] and dynamic [2] rod systems each represented by a single included cohort. Adult degenerative scoliosis cohorts (*n* = 2) and lumbosacral (L5–S1) fixation patterns were represented within the included evidence base (Table 3).

**Table 3:** Baseline characteristics of included studies

Author	Country	Study design	Sample size		Female %		Age, in years (mean ± SD or (range))		BMI, in kg/m^2^ (mean ± SD)		Smoking (*n*)		Diabetes mellitus (*n*)
			PSL	Non-PSL	PSL	Non- PSL	PSL	Non-PSL	PSL	Non-PSL	PSL	Non-PSL	PSL
Yen-Chun (2024)	Taiwan	RCS	40	275	57.5	63.6	70.0 ± 9.3	68.6 ± 6.5	26.5 ± 2.5	26.9 ± 4.1	10	33	14
Yongdi (2025)	China	RCS	27	138	70.4	65.9	55.6 ± 10.5	53.4 ± 8.0	23.9 ± 2.0	24.2 ± 2.8	7	28	4
Chen (2025)	China	RCS	63	123	46.0	55.3	66.0 ± 8.2	63.9 ± 9.4	25.4 ± 3.3	26.0 ± 2.9	8	12	7
Zan (2024)	China	RCS	17	56	47.1	57.1	60.8 ± 9.8	63.5 ± 7.7	24.4 ± 3.1	24.7 ± 3.2	3	7	NR
Yung-Hsueh (2025)	Taiwan	RCS	75	136	56.0	66.2	67.7 ± 12.1	61.5 ± 12.5	24.9 ± 6.3	25.2 ± 5.3	17	33	26
Pengyu (2025)	China	RCS	30	124	40.0	53.2	61.0 ± 5.8	60.7 ± 8.2	24.9 ± 1.6	24.8 ± 2.4	NR	NR	NR
An-Jhih (2025)	Taiwan	RCS	108	286	54.6	59.4	66.8 ± 12.0	63.6 ± 9.4	25.8 ± 3.6		NR	NR	NR
Wu (2024)	China	RCS	40	100	47.5	61.0	64.7 ± 9.3	61.9 ± 8.1	24.2 ± 2.4	25.0 ± 3.0	NR	NR	NR
Guozheng (2024)	China	RCS	24	93	70.8	54.8	61.7 ± 8.3	57.8 ± 7.4	25.6 ± 3.3	24.9 ± 3.5	6	13	5
Jingchi (2022)* [L4]	China	RCS	24	30	58.3	33.3	63.6 ± 9.9	56.8 ± 12.7	24.9 ± 3.1	25.2 ± 3.6	NR	NR	NR
Jingchi (2022)* [L5]	China	RCS	24	30	33.3	23.3	63.7 ± 8.7	58.1 ± 12.8	24.9 ± 3.3	25.1 ± 3.5	NR	NR	NR
Zan (2023)	China	RCS	52	122	59.6	70.5	65.9 ± 8.4	62.5 ± 7.3	24.6 ± 3.4	24.4 ± 3.5	4	7	8
He (2023)	China	RCS	33	57	48.5	56.1	65.5 ± 7.9	64.9 ± 7.3	NR	NR	NR	NR	NR
Kyeong (2022)	South Korea	RCS	35	78	48.6	46.2	70.6 ± 6.3	62.6 ± 11.4	25.2 ± 3.4	25.8 ± 3.3	2	6	12
Maximilian (2021)	Germany	RCS	23	23	60.9	60.9	68.5 ± 8.1	71.4 ± 10	28.6 ± 3.3	27.9 ± 5.3	NR	NR	NR
Fei (2020)	China	RCS	29	114	44.8	60.5	62.0 ± 6.7	62.0 ± 6.4	26.9 ± 2.9	25.7 ± 3.3	NR	NR	NR
Guo (2020)	China	RCS	37	180	89.2	81.7	69.1 ± 5.6	71.9 ± 8.1	23.5 ± 3.0	24.0 ± 2.7	NR	NR	NR
Leng (2020)* [≥ 6 lvls]	China	RCS	40	28	90.0	85.7	60.9 ± 7.0	61.6 ± 6.1	25.5 ± 3.7	27.1 ± 3.6	NR	NR	NR
Leng (2020)* [4–5 lvls]	China	RCS	37	32	62.2	90.6	65.1 ± 5.6	62.8 ± 5.5	25.5 ± 4.4	26.1 ± 3.3	NR	NR	NR
Cho (2018)	South Korea	RCS	17	69	58.8	72.5	69.6 ± 6.1	65.0 ± 7.4	NR	NR	NR	NR	NR
Sakai (2018)	Japan	RCS	12	40	50.0	60.0	70.4 ± 6.9	67.6 ± 10.9	24.0 ± 2.4	24.5 ± 3.8	NR	NR	NR
Matsukawa (2017)	Japan	RCS	16	335	56.3	54.0	64.8 ± 15.6	63.2 ± 13.5	26.1 ± 3.8	26.1 ± 3.1	NR	NR	NR
Li 2024	China	RCS	52	40	59.6	67.5	62.5 ± 7.9	59.7 ± 4.9	25.0 ± 3.8	24.7 ± 2.6	11	9	5
Shu 2023	China	RCS	44	171	40.9	42.7	59.3 ± 7.9	57.4 ± 7.3	24.8 ± 4.6	25.4 ± 4.3	6	15	10
Li 2023	China	RCS	55	101	61.8	61.4	60.7 ± 9.0	52.7 ± 10.6	26.8 ± 3.5	26.9 ± 3.4	8	14	4
Li 2024	China	RCS	35	209	60.0	64.6	61.0 ± 6.8	61.4 ± 8.1	25.5 ± 3.0	25.2 ± 3.7	9	45	6
Şakir Ekşi 2025	Turkey	RCS	18	100	NR	NR	62.7 ± 7.7	53.0 ± 10.2	NR	NR	NR	NR	NR
Zhou 2024	China	RCS	77	135	41.6	58.5	59.1 ± 9.60	58.20 ± 9.7	26.8 ± 4.7	26.0 ± 4.1	21	13	16
Yuya 2023	Japan	RCS	17	85	52.9	41.2	73.0 ± 10.0	69.0 ± 8.0	23.8 ± 2.5	24.5 ± 3.7	2	16	0
Wei (2021)	China	RCS	59	34	72.9	85.3	64.0 ± 6.1	62.7 ± 5.2	25.5 ± 4.2	26.4 ± 4.2	NR	NR	NR
Jinxiang (2024)	China	RCS	34	173	70.6	72.3	63.9 ± 11.3	59.0 ± 9.6	NR	NR	NR	NR	5

Author	Diabetes mellitus (*n*)	Alcohol abuse (*n*)		Hypertension (*n*)		Indication for index fusion		Type of index fusion		Surgical level		Follow-up, in months (mean ± SD)	
	Non-PSL	PSL	Non-PSL	PSL	Non-PSL	PSL	Non-PSL	PSL	Non-PSL	PSL	Non-PSL	PSL	Non-PSL
Yen-Chun (2024)	86	NR	NR	23	148	Degenerative lumbar disease		TLIF		2-level (n = 14); 3-level (n = 26)	2-level (n = 89); 3-level (n = 186)	24.0	
Yongdi (2025)	11	3	15	10	43	Degenerative spondylolisthesis (*n* = 7); spondylolytic spondylolisthesis (*n* = 20)	Degenerative spondylolisthesis (*n* = 35); spondylolytic spondylolisthesis (*n* = 103)	TLIF		L5-S1 (n = 18)	L5-S1 (n = 59)	32.9 ± 10.6	32.8 ± 12.2
Chen (2025)	21	NR	NR	29	46	Lumbar degenerative disease		PLIF		1-level between L3-S1		12.0	
Zan (2024)	NR	NR	NR	NR	NR	Lumbar disc herniation; spinal stenosis; spondylolisthesis		PLIF (n = 11); TLIF (n = 6)	PLIF (n = 29); TLIF (n = 27)	L5		12.6 (11.4–14.2)	
Yung-Hsueh (2025)	30	NR	NR	NR	NR	Degenerative spondylolisthesis (*n* = 43); spondylolytic spondylolisthesis (*n* = 16); degenerative scoliosis (*n* = 11); herniated disc (*n* = 5)	Degenerative spondylolisthesis (*n* = 95); spondylolytic spondylolisthesis(*n* = 17); degenerative scoliosis (*n* = 12); herniated disc (*n* = 12)	TLIF		L4-S1: 1-level (*n* = 102); 2-levels (*n* = 109)		34.7 ± 16.4	
Pengyu (2025)	NR	NR	NR	NR	NR	Disc herniation (*n* = 10); spondylolisthesis(*n* = 6); spinal stenosis (*n* = 14)	Disc herniation (*n* = 40); spondylolisthesis (*n* = 33); spinal stenosis (*n* = 51)	TLIF		L3/4 (*n* = 1); L4/5 (n = 21); L5/S1 (*n* = 8)	L3/4 (*n* = 12); L4/5 (*n* = 84); L5/S1 (*n* = 28)	12.0	
An-Jhih (2025)	NR	NR	NR	NR	NR	Spinal stenosis (*n* = 50); disc herniation (*n* = 7); degenerative spondylolisthesis (*n* = 81); spondylolytic spondylolisthesis (*n* = 18); degenerative scoliosis (*n* = 14)	Spinal stenosis (*n* = 86); disc herniation (*n* = 28); degenerative spondylolisthesis (*n* = 240); spondylolytic spondylolisthesis (*n* = 34); degenerative scoliosis (*n* = 24)	TLIF		1-level (*n* = 43); 2-levels (*n* = 65)	1-level (*n* = 164); 2-levels (*n* = 122)	12.0	
Wu (2024)	NR	NR	NR	NR	NR	Spinal stenosis (*n* = 17); spondylolisthesis (*n* = 10); disc herniation (*n* = 13)	Spinal stenosis (*n* = 36); spondylolisthesis (*n* = 28); disc herniation (*n* = 36)	TLIF		1-level (*n* = 25); 2-levels (*n* = 14); 3-levels (*n* = 1)	1-level (*n* = 78); 2-levels (*n* = 18); 3-levels (*n* = 4)	12.0	
Guozheng (2024)	15	5	14	7	29	Disc herniation (*n* = 11); spinal stenosis (*n* = 9); spine instability (*n* = 4)	Disc herniation (*n* = 45); spinal stenosis (*n* = 38); spine instability (*n* = 10)	Lumbar interbody fusion		NR	NR	37.5 ± 11.4	
Jingchi (2022)* [L4]	NR	NR	NR	NR	NR	Degenerative spondylolisthesis; Spinal stenosis		OLIF		1-level L4-L5		12.2 (11.0–13.5)	
Jingchi (2022)* [L5]	NR	NR	NR	NR	NR	Degenerative spondylolisthesis; Spinal stenosis		OLIF		1-level L4-L5		12.2 (11.0–13.5)	
Zan (2023)	11	2	5	38	14	Lumbar stenosis (*n* = 21); disc herniation (*n* = 9); lumbar spondylolisthesis (*n* = 12); degenerative scoliosis (*n* = 10)	Lumbar stenosis (*n* = 51); disc herniation (*n* = 38); lumbar spondylolisthesis (*n* = 30); degenerative scoliosis (*n* = 3)	PLIF		1-level (*n* = 97); 2-levels (*n* = 57); 3-levels (*n* = 12); 4-levels (*n* = 8)		12.2 ± 1.3	
He (2023)	NR	NR	NR	NR	NR	Lumbar disc herniation; spinal stenosis; spondylolisthesis; scoliosis		NR	NR	NR	NR	6.0	
Kyeong (2022)	25	NR	NR	21	36	Spinal degenerative diseases		Lumbar fusion		1-level L1-L5		6.0	
Maximilian (2021)	NR	NR	NR	NR	NR	Instability and spinal stenosis (*n* = 15); instability without spinal stenosis (*n* = 6); spondylolisthesis with spinal stenosis and instability (*n* = 1); spondylolisthesis without spinal stenosis (*n* = 1)	Instability and spinal stenosis (*n* = 7); instability without spinal stenosis (*n* = 2); spondylolisthesis with spinal stenosis and instability (*n* = 2); spondylolisthesis without spinal stenosis (*n* = 2)	Lumbar interbody fusion		L1-5 (*n* = 1); L2-5 (*n* = 8); L2-S1 (*n* = 4); L3-S1 (*n* = 9); L4-S1 (*n* = 1)	L1-5 (*n* = 1); L2-5 (*n* = 8); L2-S1 (*n* = 4); L3-S1 (*n* = 9); L4-S1 (*n* = 1)	12.0	
Fei (2020)	NR	NR	NR	NR	NR	Degenerative lumbar disease		Lumbar interbody fusion		NR	NR	12.0	
Guo (2020)	NR	NR	NR	NR	NR	Degenerative lumbosacral disease		PLIF (n = 12); TLIF (n = 25)	PLIF (n = 38); TLIF (n = 142)	Single and multilevel lumbar fusion		NR	NR
Leng (2020)* [≥ 6 lvls]	NR	NR	NR	NR	NR	NR	NR	NR	NR	Multilevel lumbar fusion		12.0	
Leng (2020)* [4–5 lvls]	NR	NR	NR	NR	NR	NR	NR	NR	NR	Multilevel lumbar fusion		12.0	
Cho (2018)	NR	NR	NR	NR	NR	Lumbar degenerative diseases		PLIF		1-level lumbar fusion		12.0	
Sakai (2018)	NR	NR	NR	NR	NR	Lumbar degenerative disease		1-level PLIF		1-level lumbar fusion		12.0	
Matsukawa (2017)	NR	NR	NR	NR	NR	Degenerative spondylolisthesis(*n* = 10); degenerative disc disease (*n* = 5); foraminal stenosis (n = 1)	Degenerative spondylolisthesis(n = 254); degenerative disc disease (*n* = 67); foraminal stenosis (*n* = 14)	1-level PLIF		L3 (*n* = 2); L4 (*n* = 8); L5 (n = 6)	L3 (*n* = 26); L4 (*n* = 168); L5 (n = 141)	25.6 ± 10.2	25.6 ± 10.2
Li 2024	5	NR	NR	12	9	Degenerative scoliosis		PLIF		Thoracic (*n* = 23); thoracolumbar (*n* = 23); lumbar (*n* = 4)	Thoracic (*n* = 15); thoracolumbar (*n* = 23); lumbar (*n* = 4)	12.0	
Shu 2023	32	NR	NR	NR	NR	Degenerative lumbar disease		Lumbar spine surgeries		NR	NR	19.4 ± 10.8	18.7 ± 11.5
Li 2023	18	NR	NR	29	37	Degenerative lumbar disease		PLIF		Levels of fixation > 4, with L4 as lowest instrumented vertebra		12.0	
Li 2024	30	9	42	11	63	Lumbar stenosis (*n* = 18); degenerative spondylolisthesis (*n* = 9); spondylolytic spondylolisthesis (*n* = 8)	Lumbar stenosis (n = 101); degenerative spondylolisthesis (*n* = 55); spondylolytic spondylolisthesis (*n* = 53)	PLIF		NR	NR	NR	NR
Şakir Ekşi 2025	NR	NR	NR	NR	NR	Spinal stenosis		L3-S1		L3-S1		35.9 ± 25.3	
Zhou 2024	14	NR	NR	NR	NR	Spinal stenosis		Lumbar interbody fusion		L5–S1		27.5 ± 9.5	28.1 ± 12.6
Yuya 2023	11	NR	NR	NR	NR	Foraminal stenosis (*n* = 38); lumbar canal/foraminal stenosis (*n* = 29); isthmic spondylolisthesis (*n* = 20); disc herniation (*n* = 4); degenerative spondylolisthesis (*n* = 8)	Foraminal stenosis (*n* = 38); lumbar canal/foraminal stenosis (*n* = 29); isthmic spondylolisthesis (*n* = 20); disc herniation (*n* = 4); degenerative spondylolisthesis (*n* = 8)	PLIF (*n* = 11); TLIF (*n* = 6)	PLIF (*n* = 56); TLIF (*n* = 29)	NR	NR	6.0	
Wei (2021)	NR	NR	NR	NR	NR	Lumbar degenerative disease		Interbody fusion		NR	NR	38.4 ± 16.0	
Jinxiang (2024)	12	NR	NR	14	44	Degenerative lumbar disease		Interbody fusion		1-level lumbar fusion		12.0	

Summary of demographic and baseline characteristics for all studies included in the meta-analysis. Columns report study author and publication year, country of study origin, study design, sample size, sex distribution, age distribution, BMI, smoking, diabetes mellitus, alcohol abuse, hypertension, indication for fusion, type of fusion, vertebral levels involved, and duration of follow-up. *PSL* pedicle screw loosening, *RCS* retrospective cohort study, *SD* standard deviation, *NR* not reported, *BMI* body mass index, *HTN* hypertension, *ALIF* anterior lumbar interbody fusion, *OLIF* oblique lumbar interbody fusion, *PLIF* posterior lumbar interbody fusion, *TLIF* transforaminal lumbar interbody fusion, *XLIF* extreme lumbar interbody fusion, *PLF* posterolateral fusion, *n* number of patients. *Data for Jingchi et al. (2022) and Leng et al. (2020) presented in two rows as per original authors’ grouping

### Bone-based imaging metrics: inter-group comparisons

**Fig. 2:**
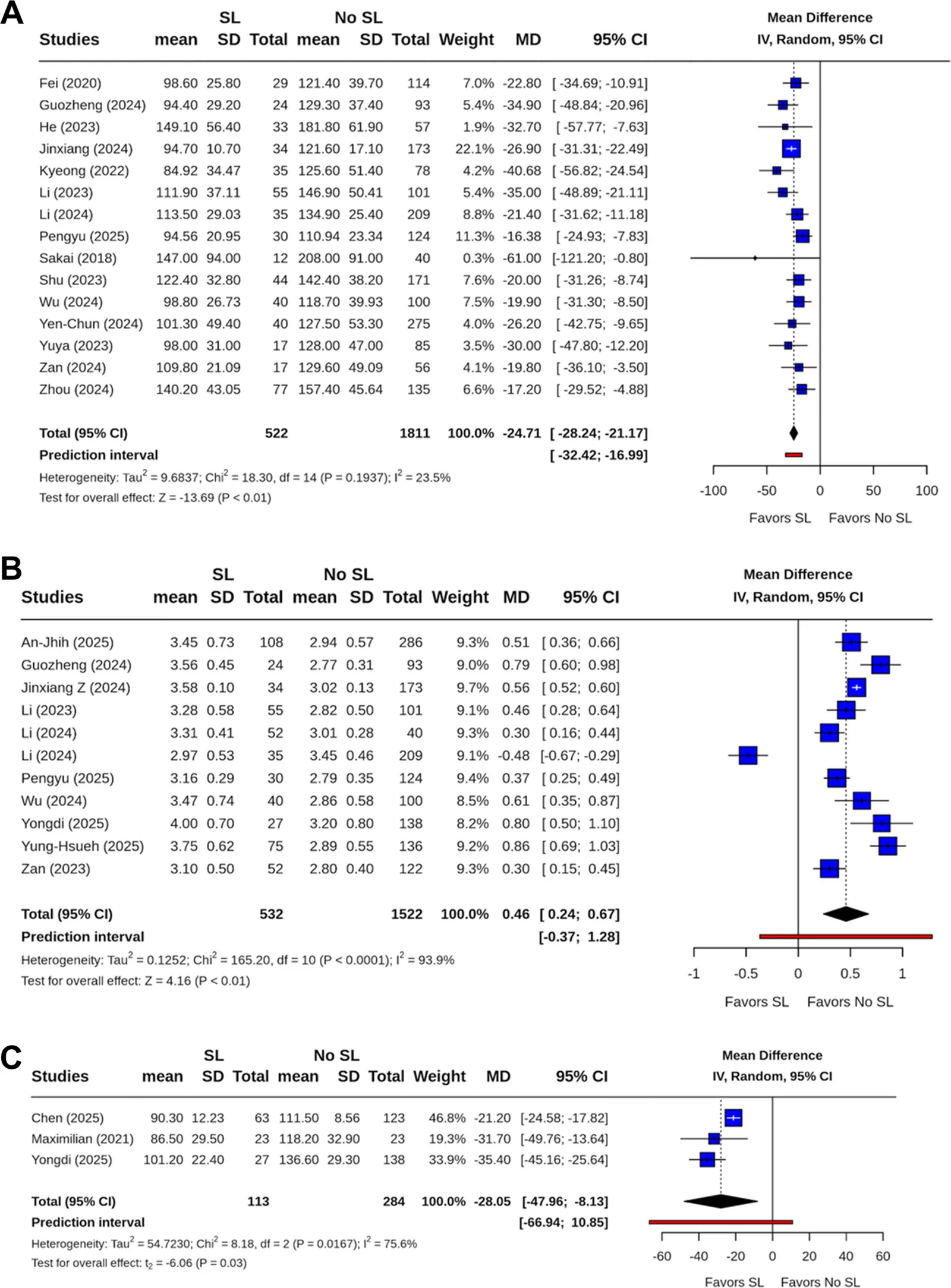
Hounsfield units, VBQ, and CT-BMD. Forest plots (random effects model) comparing (**A**) Hounsfield units (HU), (**B**) vertebral bone quality (VBQ), and (**C**) CT-derived bone mineral density (CT-BMD) between patients with (‘SL’) and without screw loosening (‘No SL’). Individual study estimates are shown as squares proportional to study weight. Horizontal lines indicate 95% confidence intervals. Diamond represents pooled estimate for overall effect. IV = inverse variance; MD = mean difference

Fifteen studies [5, 27, 29, 30, 35, 39, 41, 56, 62, 65, 75, 76, 78, 79, 81] reported CT-based Hounsfield unit measurements, comprising 2333 patients, of whom 22.4% (522/2333) developed screw loosening. Patients with PSL demonstrated significantly lower Hounsfield unit values compared to those without (MD: −24.71; 95% CI: −28.24**–**−21.17; *P* < 0.01; I^2^ = 23.5%) (Fig. 2A). The direction and significance of effect remained stable on leave-one-out sensitivity analysis. Vertebral cancellous measurement was the predominant protocol (*n* = 8), with trajectory-based (*n* = 2) and pedicle-focused (*n* = 1) approaches sparsely represented, with the remainder unclassifiable. Formal subfamily pooling and moderator analyses were therefore not undertaken.

Eleven studies [6, 27, 30, 39, 41, 42, 44, 65, 68, 76, 78] reported MRI-based VBQ measurements in 2054 patients, with a total PSL incidence of 25.9% (532/2054). VBQ scores were significantly higher in patients with PSL compared to those without (MD: 0.46; 95% CI: 0.24–0.67; *P* < 0.01; I^2^ = 93.9%) (Fig. 2B). The direction and significance of effect remained stable on leave-one-out sensitivity analysis. The high observed heterogeneity (I^2^ = 93.9%) partly reflects the inclusion under a unified VBQ heading of three protocol variants (Table 1): standard L1–L4 VBQ, sacral VBQ, and PBQ. Standard L1–L4 VBQ studies showed tightly concordant directional effects, whereas PBQ-based studies showed conflicting directions. Baujat inspection (Supplementary Fig. 16) confirmed PBQ-based studies as among the most influential heterogeneity contributors.

Three studies [5, 43, 68] reported CT-BMD values in 397 patients. The overall incidence of PSL in this group was 28.5% (113/397). Patients with PSL had significantly lower CT-BMD compared to those without (MD: −28.05; 95% CI: −47.96**–**−8.13; *P* < 0.05; I^2^ = 75.6%) (Fig. 2C). Findings were robust on leave-one-out sensitivity analysis.

Ten studies [5, 8, 19, 35, 37, 41, 42, 44, 69, 79] reported DEXA T-scores in 1652 patients, among whom 31.2% (516/1652) developed PSL. DEXA T-scores were significantly lower in PSL cohorts (MD: −0.18; 95% CI: −0.32**–**−0.05; *P* < 0.01; I^2^ = 25.8%) (Supplementary Fig. 1). Results were stable on leave-one-out sensitivity analysis.

### Diagnostic accuracy of bone-based metrics

**Fig. 3:**
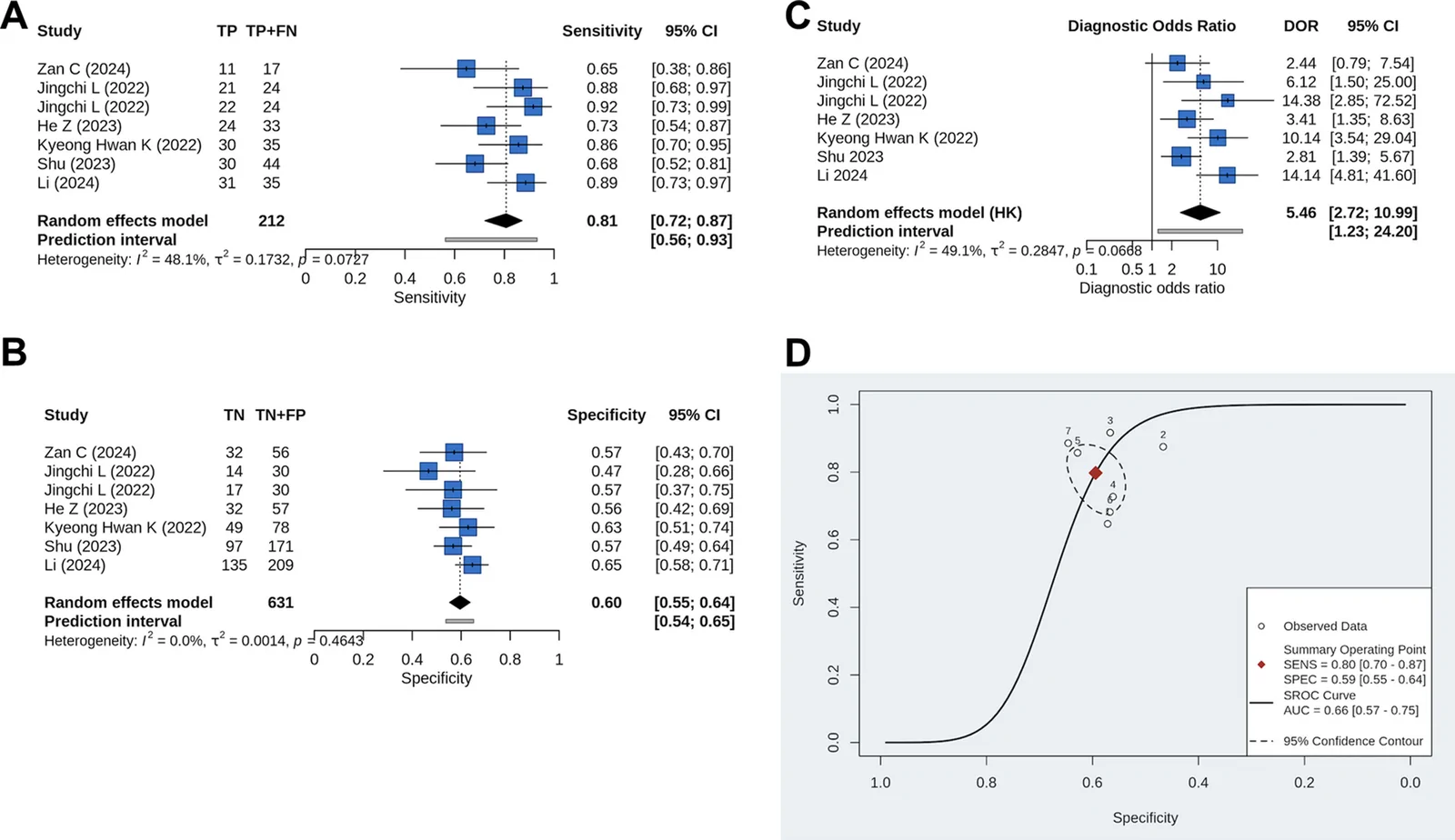
Diagnostic performance of Hounsfield units. Forest plots (random effects model) reporting (**A**) sensitivity, (**B**) specificity, and (**C**) diagnostic odds ratio (DOR) for Hounsfield unit scores for identifying screw loosening. Individual study estimates are shown as squares proportional to study weight. Horizontal lines indicate 95% confidence intervals. Diamond represents pooled estimate for overall effect. (**D**)Summary receiver operating characteristic (SROC) curve showing the overall diagnostic accuracy of HU, with the summary operating point (pooled sensitivity and specificity), 95% confidence region, and the area under the curve (AUC). IV = inverse variance; GLMM = generalized linear mixed model

Six studies [6, 35, 40, 41, 62, 79] reported diagnostic accuracy data for Hounsfield units. The Youden-derived Hounsfield unit threshold reported across the included studies clustered around 128.8 and is presented descriptively for reference at the study-level (this value is not a meta-analytically pooled cutoff) (Table 1). Pooled sensitivity was 0.81 (95% CI: 0.72–0.87, I^2^ = 48.1%) and specificity was 0.60 (95% CI: 0.55–0.64, I^2^ = 0.0%). The pooled dOR was 5.46 (95% CI: 2.72–10.99; I^2^ = 49.1%) (Fig. 3A-C). The SROC curve (Fig. 3D) demonstrated a pooled AUC of 0.71 (95% CI: 0.67–0.76; I^2^ = 53.3%) (Supplementary Fig. 2). Full diagnostic metrics are provided (Table 4).

**Table 4:** Pooled diagnostic metrics

Metric	Cutoff	Sensitivity (95% CI)	Specificity (95% CI)	Diagnostic odds ratio (95% CI)	Positive likelihood ratio (95% CI)	Negative likelihood ratio (95% CI)
HU	128.8	0.81 (0.72–0.87)	0.60 (0.55–0.64)	5.46 (2.72–10.99)	1.99 (1.68–2.32)	0.32 (0.21–0.48)
VBQ	3.18	0.83 (0.78–0.87)	0.68 (0.61–0.74)	10.15 (6.02–17.11)	2.59 (2.12–3.22)	0.25 (0.19–0.33)
CT-BMD	103.6	0.83 (0.75–0.89)	0.62 (0.46–0.76)	8.83 (0.93–84.24)	2.27 (1.52–3.67)	0.25 (0.14–0.46)

Summary of diagnostic accuracy metrics for imaging-based bone quality measures in predicting pedicle screw loosening. Study-level Youden-derived cut-off values are representative values shown descriptively and are not meta-analytically pooled; pooled sensitivity, specificity, diagnostic odds ratio (DOR), positive likelihood ratio (PLR), and negative likelihood ratio (NLR) are model-derived from random-effects meta-analyses and presented with 95% confidence intervals (CIs). *HU* Hounsfield units, *VBQ* vertebral bone quality, *CT-BMD* computed tomography-derived bone mineral density

**Fig. 4:**
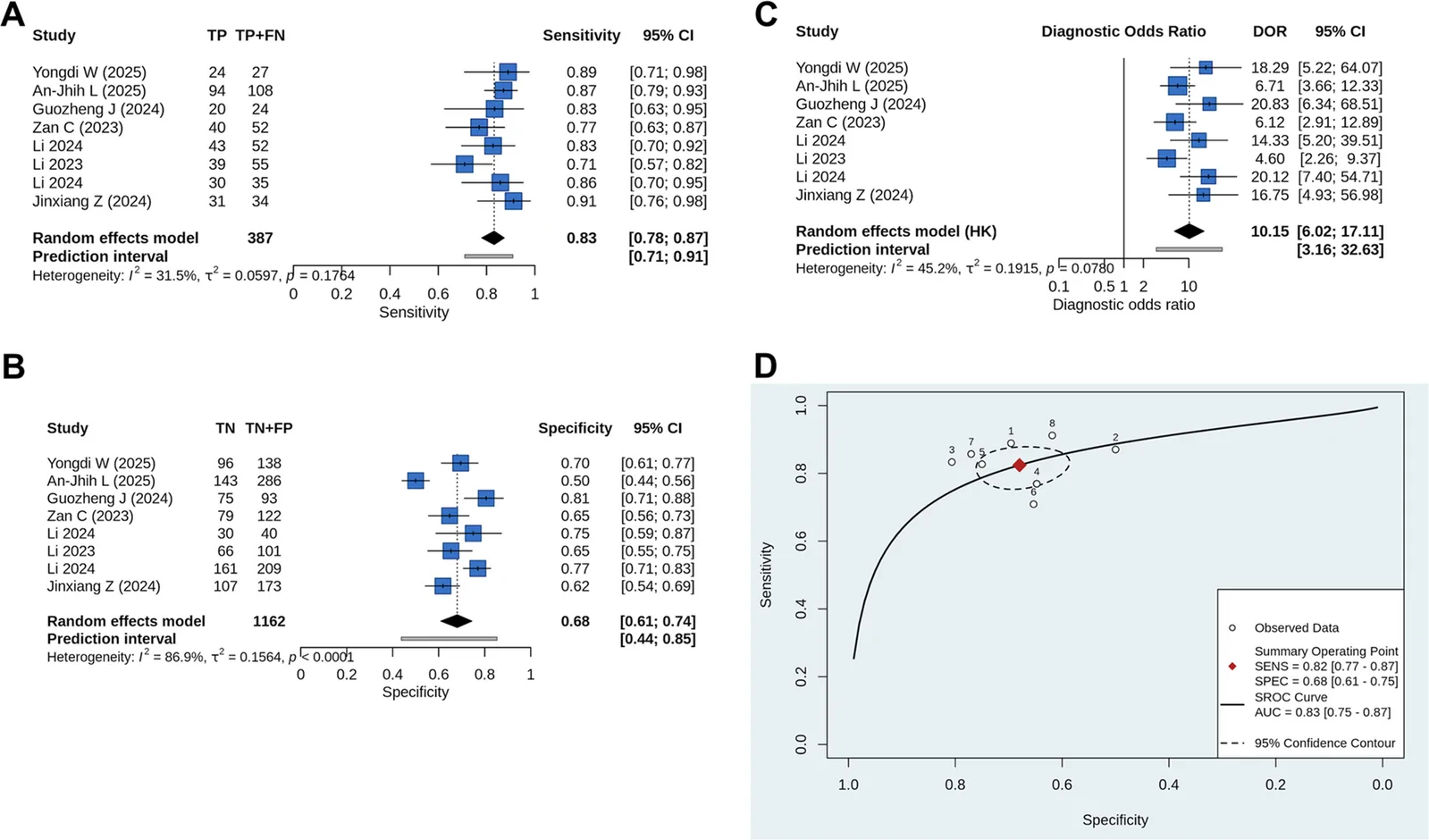
Diagnostic performance of VBQ. Forest plots (random effects model) reporting (**A**) sensitivity, (**B**) specificity, and (**C**) diagnostic odds ratio (DOR) for vertebral bone quality (VBQ) for identifying screw loosening. Individual study estimates are shown as squares proportional to study weight. Horizontal lines indicate 95% confidence intervals. Diamond represents pooled estimate for overall effect. (**D**) Summary receiver operating characteristic (SROC) curve showing the overall diagnostic accuracy of VBQ, with the summary operating point (pooled sensitivity and specificity), 95% confidence region, and the area under the curve (AUC). IV = inverse variance; GLMM = generalized linear mixed model

Eight studies [6, 30, 39, 41, 42, 44, 68, 78] reported diagnostic performance for VBQ. The Youden-derived VBQ threshold reported across the included studies clustered around 3.18 and is presented descriptively for reference at the study-level (this value is not a meta-analytically pooled cutoff) (Table 1). Pooled sensitivity was 0.83 (0.78–0.87, I^2^ = 31.5%) and specificity was 0.68 (0.61–0.74, I^2^ = 86.9%). The pooled dOR was 10.15 (95% CI: 6.02–17.11; I^2^ = 45.2%) (Fig. 4A-C). The SROC curve (Fig. 4D) yielded a pooled AUC of 0.76 (95% CI: 0.74–0.79; I^2^ = 0.0%) (Supplementary Fig. 3).

**Fig. 5:**
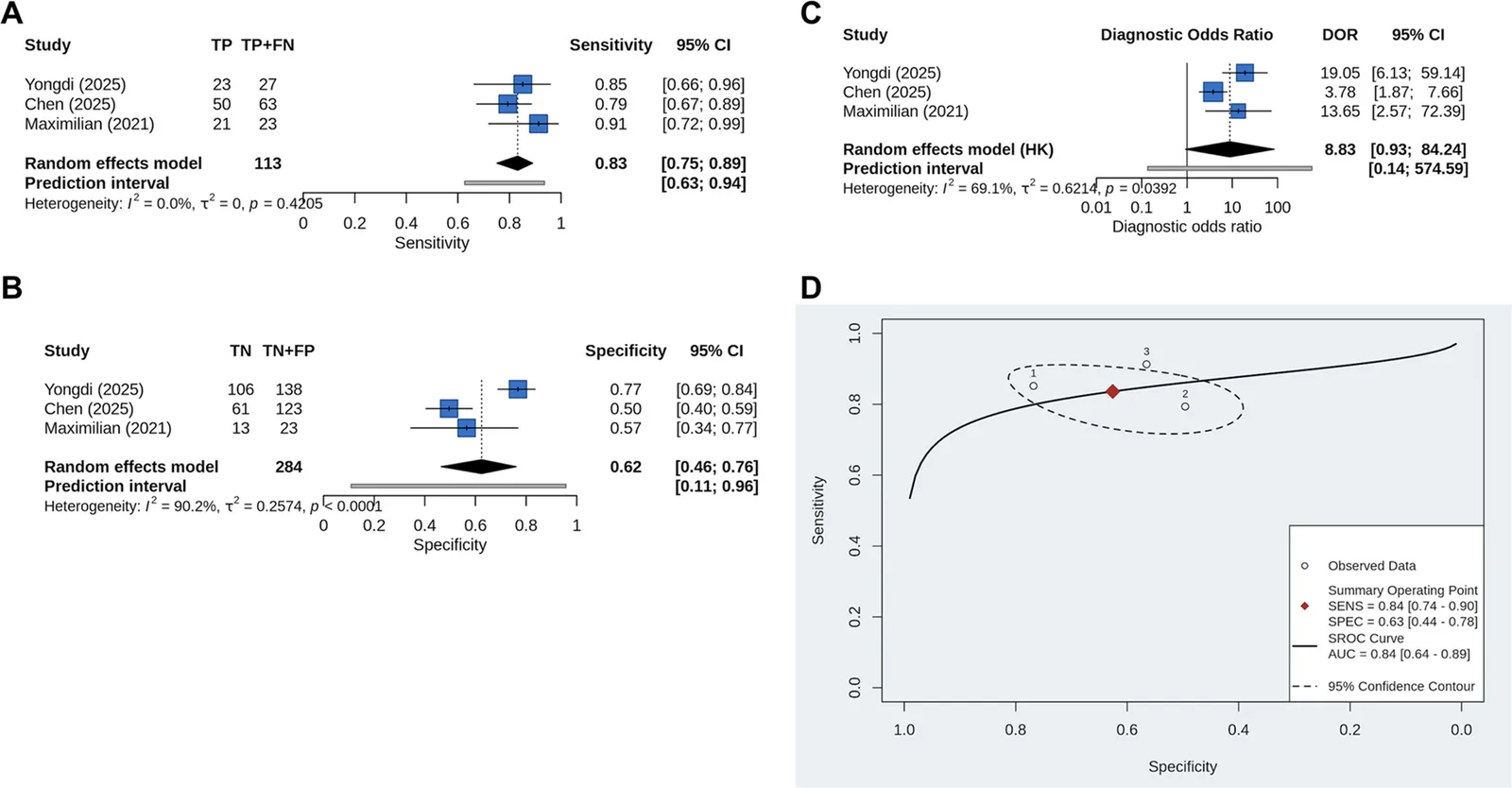
Diagnostic performance of CT-BMD. Forest plots (random effects model) reporting (**A**) sensitivity, (**B**) specificity, and (**C**) diagnostic odds ratio (DOR) for CT-derived bone mineral density (CT-BMD) for identifying screw loosening. Individual study estimates are shown as squares proportional to study weight. Horizontal lines indicate 95% confidence intervals. Diamond represents pooled estimate for overall effect. **D** Summary receiver operating characteristic (SROC) curve showing the overall diagnostic accuracy of BMD, with the summary operating point (pooled sensitivity and specificity), 95% confidence region, and the area under the curve (AUC). IV = inverse variance; GLMM = generalized linear mixed model

Three studies [5, 43, 68] reported diagnostic performance of CT-BMD. The Youden-derived CT-BMD threshold reported across the included studies clustered around 103.6 and is presented descriptively for reference at the study-level (this value is not a meta-analytically pooled cutoff) (Table 1). Pooled sensitivity was 0.83 (95% CI: 0.75–0.89, I^2^ = 0.0%) and specificity was 0.62 (95% CI: 0.46–0.76, I^2^ = 90.2%). The pooled dOR was 8.83 (95% CI: 0.93–84.24; I^2^ = 69.1%) (Fig. 5A-C). The SROC curve (Fig. 5D) demonstrated a pooled AUC of 0.76 (95% CI: 0.56–0.97; I^2^ = 76.9%) (Supplementary Fig. 4). The high specificity heterogeneity (I^2^ = 90.2%) reflects substantive protocol differences across the included studies, ranging from quantitative CT at L5 to opportunistic CT-BMD screening at variable lumbar levels. With only three studies, this spread reflects protocol non-comparability rather than a separable methodological signal, and CT-BMD diagnostic estimates are interpreted accordingly as preliminary and protocol-sensitive. Across all three metrics, pooled sensitivity and specificity are conditional on a mixed, non-uniform reference standard: PSL was adjudicated on plain radiographs alone in approximately 45% of studies, on CT alone in 34%, and on combined radiograph-and-CT or MRI in the remainder, so the pooled operating characteristics reflect a composite radiographic endpoint rather than uniform discrimination. Descriptively, radiograph-only (45%) and CT-adjudicated (34%) cohorts differ in ascertainment: CT resolves early peri-screw lucency that radiographs miss, so radiograph-based cohorts likely under-ascertain incipient loosening and the two are not exchangeable reference standards. Because modality also co-varies with the index test, geography, and surgical era, these strata cannot be cleanly separated into an unconfounded modality-stratified estimate; per-study modality, definition, timing, and threshold are itemized in Table 1 rather than pooled by modality.

### Paraspinal muscle metrics

Psoas size metrics showed the most consistent association with PSL. Pooled analysis of two studies [69, 81] demonstrated significantly lower psoas rtCSA in the PSL group (MD −0.05; 95% CI: −0.09–−0.01; *P* < 0.05; I^2^ = 15.0%), with directional concordance in 85.7% (6/7) of comparisons (Supplementary Fig. 5). Similarly, psoas rfCSA was significantly lower in PSL cohorts (MD −0.06; 95% CI: −0.10–−0.02; *P* < 0.05; I^2^ = 0.0%), with concordant directionality across 100.0% (7/7) of comparisons (Supplementary Fig. 6). Psoas MFI, reported in two studies [37, 69], was not significantly associated with PSL (MD 0.02; 95% CI: −0.17–0.21; *P* = 0.85; I^2^ = 0.0%) (Supplementary Fig. 7).

No erector spinae metric demonstrated a significant association with PSL. For erector spinae rtCSA, pooled analysis of two studies [69, 81] demonstrated no significant difference (MD −0.02; 95% CI: −0.30–0.17; *P* = 0.84; I^2^ = 58.3%) (Supplementary Fig. 8). Similarly, pooled erector spinae rfCSA analysis of three studies [37, 69, 81] showed no significant difference (MD −0.03; 95% CI: −0.14–0.09; *P* = 0.66; I^2^ = 75.5%) (Supplementary Fig. 9). Erector spinae MFI was also not significantly associated with PSL (MD −0.02; 95% CI: −0.04–0.05; *P* = 0.15; I^2^ = 36.0%) (Supplementary Fig. 10).

Across all multifidus metrics, pooled effects were small and non-significant. Multifidus rtCSA (MD −0.01; 95% CI: −0.06–0.05; *P* = 0.88; I^2^ = 58.3%) (Supplementary Fig. 11), rfCSA (MD 0.00; 95% CI: −0.04–0.03; *P* = 0.93; I^2^ = 0.0%) (Supplementary Fig. 12), and MFI (MD 0.16; 95% CI: −0.25–0.57; *P* = 0.44; I^2^ = 59.6%) (Supplementary Fig. 13) all demonstrated directionally negative but non-significant trends.

Paraspinal muscle metrics are presented as an exploratory analysis given small per-cell study counts and residual heterogeneity in normalization and segment of measurement.

### Clinical outcomes

Fusion was reported in five studies [8, 27, 44, 69, 81] and associated with a significantly lower risk in PSL cohorts (RR: 0.84; 95% CI: 0.71–0.98; *P* < 0.05; I^2^ = 87.3%) (Supplementary Fig. 14).

Preoperative ODI scores, reported in seven studies [8, 27, 28, 39, 41, 42, 68], were significantly higher in PSL cohorts (MD: 2.07; 95% CI: 0.84–3.29; *P* < 0.01; I^2^ = 0.0%) (Supplementary Fig. 15 A), and postoperative ODI scores, reported in eight studies [8, 27, 28, 39, 41, 42, 68, 81], remained significantly higher (MD: 1.15; 95% CI: 0.26–2.04; *P* = 0.01; I^2^ = 0.0%) (Supplementary Fig. 15B).

Seven studies [8, 27, 28, 39, 41, 42, 68] reported preoperative VAS back pain, with no significant difference between groups (MD: 0.10; 95% CI: −0.06–0.26; *P* = 0.23; I^2^ = 27.4%) (Supplementary Fig. 15C). Eight studies [8, 27, 28, 39, 41, 42, 68, 81] reported postoperative VAS back pain, which was associated with significantly higher scores in PSL cohorts (MD: 0.39; 95% CI: 0.09–0.70; *P* < 0.05; I^2^ = 79.1%) (Supplementary Fig. 15D).

### Clinical interpretability: Fagan nomogram plots

**Fig. 6:**
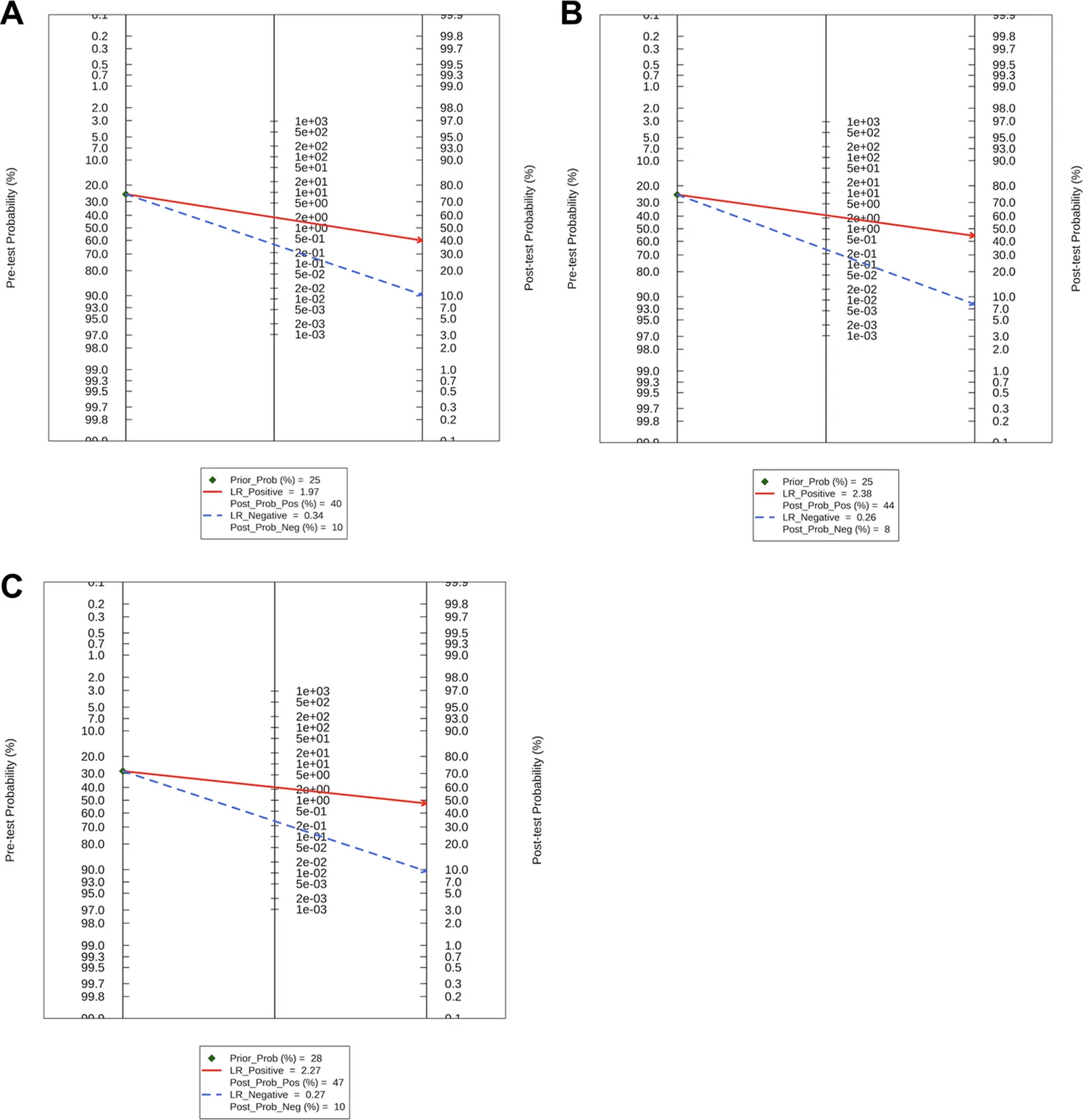
Fagan nomograms for Hounsfield units, VBQ, and CT-BMD. Fagan plots demonstrating the change from pre-test to post-test probability of screw loosening using pooled likelihood ratios for (**A**) Hounsfield units (HU), **B** vertebral bone quality (VBQ), and (**C**) CT-derived bone mineral density (CT-BMD). Solid red lines indicate the post-test probability following a positive test result, while dashed blue lines indicate the post-test probability following a negative test result

Fagan nomograms were constructed to contextualize diagnostic performance (Fig. 6). Assuming a 25.0% pre-test probability of PSL, a positive test increased post-test probability to 40.0% for Hounsfield units, 44.0% for VBQ, and 47.0% for CT-BMD, while a negative test reduced post-test probability to 10.0%, 8.0%, and 10.0%, respectively.

### Influence analysis, threshold effects, and meta-regression

Baujat plots were used to identify studies contributing most to heterogeneity and influence on pooled estimates for VBQ, CT-BMD, fusion, and postoperative VAS back pain (Supplementary Fig. 16). Threshold-effect assessment demonstrated no significant threshold effects for Hounsfield units (ρ = 0.04; *P* = 0.94; *n* = 7), VBQ (ρ = 0.29; *P* = 0.49; *n* = 8), or CT-BMD (ρ = −0.50; *P* = 0.67; *n* = 3), although inference for CT-BMD was limited by small study numbers and should be considered exploratory and supportive rather than definitive.

Exploratory meta-regression including metric type (Hounsfield unit, VBQ, CT-BMD) as a moderator (k = 29 metric-level observations: 15 HU, 11 VBQ, and 3 CT-BMD between-group comparisons, so multi-family studies contribute more than once) showed no significant difference in metric-level effect estimates across indicators (QM = 4.94; df = 2; *P* = 0.08). Because this model does not fully account for multiple metric families per study, its standard errors are potentially anti-conservative and the near-threshold *P* value should not be overinterpreted as a true between-metric difference.

### Publication bias, risk of bias, and certainty of evidence

Funnel plot inspection demonstrated no major asymmetry (Supplementary Fig. 16). Deeks’ funnel plot asymmetry test showed no evidence of publication bias for Hounsfield units (*P* = 0.15). Overall risk of bias was moderate in 28 studies and serious in one study (Supplementary Table 2). Certainty of evidence for primary diagnostic metrics was moderate, with downgrading primarily driven by risk of bias (Supplementary Table 3A–C).

## Discussion

This systematic review and meta-analysis synthesizes the current evidence on opportunistic imaging-derived bone quality and paraspinal muscle metrics for predicting PSL after degenerative lumbar fusion. Across 29 observational studies encompassing 4711 patients, a coherent and biologically plausible signal emerged: patients who developed PSL demonstrated poorer preoperative bone quality across multiple imaging modalities, including lower CT-based Hounsfield units, lower CT-BMD, lower DEXA T-scores, and higher MRI-based VBQ scores, alongside reduced psoas muscle CSA where assessed. In parallel, diagnostic accuracy analyses demonstrated moderate but consistent discrimination for CT- and MRI-based bone metrics, with broadly comparable performance across modalities. Collectively, these findings support the interpretation that PSL reflects a substrate-driven vulnerability at the bone-screw interface, which can be identified preoperatively using routinely acquired imaging, but not deterministically predicted by any single metric.

### Bone quality as a substrate-level determinant of screw loosening

The inter-group comparisons were directionally consistent across all bone-based metrics and align with established biomechanical principles. Lower Hounsfield unit values, lower CT-BMD, and lower DEXA T-scores in the loosening cohort reflect reduced trabecular density and altered cortical-cancellous architecture, which together diminish screw purchase and resistance to cyclic toggling and pullout forces [31, 75, 76]. This relationship is supported by recent finite element and trajectory-based studies demonstrating that lower local Hounsfield unit values are associated with reduced pullout strength under cyclic loading conditions, while trajectory optimisation toward higher density bone improves fixation strength and mechanical flexibility [31, 80]. Vertebral cancellous, trajectory-based, and pedicle-focused Hounsfield measurements are not biomechanically interchangeable. Vertebral cancellous Hounsfield units function as a global substrate surrogate, whereas the pedicle and juxtapedicular corridor more directly represent the true screw-bone interface – trajectory-based Hounsfield units sit between these in conceptual specificity. As vertebral cancellous measurement predominated in the included evidence base, pooled Hounsfield unit most closely approximate global substrate-level vulnerability rather than local interface biomechanics. Trajectory- and pedicle-focused Hounsfield units warrant prioritization in future harmonization efforts. In parallel, recent work by Hiyama et al. [24, 25] has demonstrated that simplified Hounsfield unit measurement approaches and CT-based triage strategies can be feasibly implemented in routine spine surgery workflows, with reasonable concordance to DEXA-based assessment and clinically actionable thresholds for identifying patients requiring further osteoporosis evaluation. Over time, repetitive physiological load may exceed the reduced local failure threshold, leading to progressive peri-screw resorption, radiolucent halo formation, and eventual loss of fixation stability [73]. Importantly, this process does not require technical error but rather reflects the interaction between applied mechanical demands and local bone anchorage capacity, rather than either domain in isolation.

MRI-based VBQ demonstrated a similar directional association, consistent with its conceptual basis as an indirect marker of trabecular deterioration via marrow fat infiltration [7]. However, heterogeneity was substantially higher for VBQ than for CT-based metrics, in contrast to Hounsfield units which demonstrated lower heterogeneity and greater stability of effect estimates. This likely reflects the variability in MRI acquisition characteristics, field strength, sequence parameters, ROI conventions, and population-level differences in marrow composition and region-specific adaptations (such as sacral or pedicle-based variants), as well as reproducibility challenges highlighted in recent validation and systematic review studies of both Hounsfield units and VBQ-based metrics [16, 18, 63, 70]. In particular, pooling standard L1–L4 VBQ alongside sacral and pedicle-based PBQ variants is a primary contributor to inter-group heterogeneity, with PBQ-based studies showing inconsistent directional behavior [45]. Future syntheses would benefit from separating these variants where study counts permit. This variability likely explains why VBQ performs comparably in discrimination despite wide dispersion in effect size estimates. In contrast, evidence for CT-BMD remains limited, with only three contributing studies and wide confidence intervals around diagnostic estimates, indicating substantial statistical uncertainty and limiting confidence in pooled diagnostic performance.

Rather than representing an isolated technical failure, PSL reflects a progressive loss of fixation stability that arises when repetitive mechanical demands exceed the anchorage capacity of compromised bone. Bone quality metrics do not “cause” loosening but rather, they identify a vulnerability state in which otherwise acceptable construct mechanics are less tolerant of error. Construct-level context further conditions interpretation of these pooled estimates. Across four construct-level moderators with established biomechanical relevance to PSL, the empirical evidence base is asymmetric in ways that constrain the portability of pooled estimates. Fusion length: short-segment constructs dominate, while multilevel constructs are sparsely represented, and pooled estimates may not transfer to long-segment deformity correction. Lumbosacral fixation: L5–S1 constructs are represented in a subset of cohorts, but pelvic fixation is absent, and behavior at the sacropelvic junction may diverge from purely lumbar fixation. Construct rigidity: most included cohorts used rigid constructs, while semi-rigid and dynamic rod systems were each represented by only a single cohort, leaving an important gap [30]. Surveillance modality: adjudication varied between radiograph, CT, and combined approaches with non-random distribution across studies, and CT surveillance is generally more sensitive for early peri-screw lucency. Pooled estimates most directly reflect risk in short-segment rigid lumbar fixation under mixed surveillance and should be recalibrated rather than transposed when applied to deformity, lumbopelvic, or dynamic construct populations.

### Diagnostic performance: risk stratification rather than determinism

Across diagnostic models, CT- and MRI-derived bone metrics demonstrated moderate discriminatory accuracy (pooled AUCs ranged from 0.71 to 0.76). Discrimination in this range supports the use of these measures for identifying patients in whom fixation stability is less tolerant of cyclic loading, rather than for definitive prediction of loosening in isolation. Sensitivity was generally higher than specificity, particularly for Hounsfield units, resulting in likelihood ratios that favor risk capture over definitive exclusion. This profile is appropriate for preoperative risk stratification but insufficient for standalone decision-making. Fagan nomogram analyses further contextualize this point. Assuming a pre-test probability of 25.0%, positive tests increased post-test probability to approximately 40.0–47.0% across metrics, while negative tests reduced it to 8.0–10.0%. These shifts are clinically meaningful but not decisive, underscoring that imaging-derived bone quality metrics primarily refine preoperative risk estimates and inform vigilance and construct strategy, rather than functioning as binary tests for fixation failure. In practice, whether biological vulnerability translates into fixation failure remains contingent on construct mechanics, screw trajectory and cortical engagement, implant decisions, fusion length, sagittal correction, and postoperative loading [38]. Notably, meta-regression did not identify statistically significant differences in diagnostic performance between Hounsfield units, VBQ, and CT-BMD, supporting the interpretation that these measures capture overlapping aspects of fixation-relevant bone competence rather than distinct pathological processes. This positions these metrics, Hounsfield units in particular, as instruments of preoperative risk stratification rather than deterministic prediction [53].

### Thresholds, heterogeneity, and the limits of portability

Threshold-effect analyses did not demonstrate significant cut-off–driven heterogeneity for Hounsfield units or VBQ, suggesting that inter-study variability is not primarily explained by threshold selection alone. However, the descriptive Hounsfield unit and VBQ reference values reported here are hypothesis-generating and fixation-specific, not universally applicable clinical cutoffs. Hounsfield unit thresholds vary materially with scanner parameters, reconstruction kernel, and ROI selections [65, 76], and VBQ remains reproducibility-sensitive across MRI field strengths, sequences, and ROI conventions [16, 18, 36], with S1- and pedicle-based variants further altering threshold structure within the same biological domain [42, 45]. VBQ behavior also differs in dynamic and semirigid fixation cohorts [30], reinforcing that thresholds derived in one fixation context should not be transposed to another. Rather than pursuing a single “optimal” threshold, future work should emphasize local calibration within standardized acquisition and reporting frameworks, ideally embedded within multivariable models that incorporate construct-level modifiers.

The pooled reference values identified in this analysis should also be interpreted in the context of established osteoporosis screening thresholds. For example, commonly cited Hounsfield unit cut-offs for low bone density in the lumbar spine are typically in the range of 110–135 units [54, 59], while VBQ thresholds associated with reduced bone quality have been reported around 3.0–3.5 in degenerative populations [12, 16]. Similarly, CT-based volumetric bone density thresholds used in osteoporosis assessment often fall below approximately 120 mg/cm^3^ [77]. The descriptive study-level values observed in this study (Hounsfield units ≈ 128.8, VBQ ≈ 3.18, CT-BMD ≈ 103.6) are broadly consistent with these low bone quality or osteopenic ranges. However, these PSL-oriented thresholds reflect fixation-specific rather than systemic bone disease and should not be interpreted as interchangeable with diagnostic osteoporosis criteria. Their primary value lies in identifying relative vulnerability to screw-bone interface failure within surgical populations.

### Sarcopenia and muscle reserve as complementary vulnerability signals

Among paraspinal muscle metrics, psoas CSA demonstrated the most consistent, albeit limited, association with PSL, whereas posterior paraspinal muscles and fatty infiltration indices showed heterogeneous and largely non-significant effects. This pattern suggests that global muscle reserve, rather than segment-specific degeneration alone, may act as a co-marker of fixation vulnerability. Mechanistically, reduced psoas size may reflect sarcopenia, frailty, and diminished dynamic stabilization, potentially amplifying cyclic micro-motion across instrumented segments and impairing postoperative load sharing [14, 23, 26, 46]. However, the current evidence base is limited to a small number of studies and clustered comparisons, and variable normalization strategies. Interpretation is further limited by heterogeneity in normalization methods (such as disc area versus vertebral body normalization), which may influence comparability of effect estimates across studies. Given the small number of contributing studies, formal stratified analysis by normalization approach was not feasible. As such, muscle metrics should be interpreted as hypothesis-generating adjuncts rather than established or independently predictive markers until validated in larger, methodologically standardized cohorts.

### Clinical implications: decision support, not deterministic prediction

From a clinical standpoint, these findings support the use of imaging-derived bone quality metrics as decision-support tools that translate qualitative concerns (“this looks osteoporotic”) into reproducible, preoperative risk signals. Identification of reduced bone competence should prompt proportionate interface-protective strategies, including meticulous preservation of cortical purchase, optimization of screw trajectory and length, consideration of larger or more cortical-engaging implants, and escalation of fixation strategies in higher-risk constructs where appropriate. Crucially, these metrics should not be framed as mandates for specific interventions in isolation. Rather, they identify vulnerability states in which construct design and technique become less tolerant of error, allowing surgeons to rationally modulate risk rather than attempt deterministic prediction. This decision-support framing does not rest on demonstrated outcome modification: no included study showed that altering management on these metrics reduces PSL. Reduced bone competence therefore identifies a lower-tolerance interface that should prompt structured bone-health evaluation and explicit construct-planning discussion, not directly justify cement augmentation, altered fixation, or osteoporosis pharmacotherapy absent a studied interventional pathway [17, 35, 57].

### Limitations and future directions

Several limitations should be considered when interpreting these findings. First, the available evidence base is largely retrospective and observational, with substantial variability in how PSL was defined, detected, and surveilled across studies. Radiographic criteria ranged from static halo sign assessment to longitudinal evaluation of progressive radiolucency, and follow-up duration was inconsistent, limiting the ability to distinguish early mechanical settling from true fatigue-related anchorage failure. These factors likely contribute to inter-study heterogeneity and may bias effect estimates toward later-detected events. Restriction to English language studies was applied at the database search stage and non-English records were not screened at the abstract level. Given the substantial volume of relevant spine literature published in Chinese, Japanese, Korean, and continental European journals, it is likely that some eligible cohorts were excluded on language grounds alone, although the precise number could not be quantified. This restriction, together with the predominance of single country retrospective cohorts may particularly limit the generalizability of pooled estimates to Asian and continental European surgical populations. In addition, omission of Scopus, Web of Science, and regional databases may have missed records not indexed in PubMed, Embase, or CENTRAL, further limiting search sensitivity despite reference-list screening.

Second, heterogeneity in surgical technique and construct design was considerable. Screw trajectory, length, diameter, degree of cortical engagement, fusion length, and alignment correction were variably reported and infrequently adjusted for, constraining causal inference. Given PSL reflects the cumulative effect of cyclic loading acting on local fixation strength, unmeasured differences in construct mechanics may partially confound observed associations between imaging-derived substrate metrics and loosening risk. In addition, these factors could not be formally evaluated in meta-regression due to inconsistent reporting, limiting assessment of their potential moderating effects.

Third, although bone-based imaging metrics were consistently associated with PSL, measurement protocols varied across modalities and centers. Differences in CT acquisition parameters, ROI selection, MRI field strength, and VBQ calculation approaches may limit direct comparability and reduce the portability of proposed thresholds. Diagnostic accuracy estimates therefore reflect average discriminative performance rather than center-specific calibration. Several included studies derived and evaluated index test thresholds within the same cohort, a recognized source of optimism bias that likely inflates pooled operating characteristics relative to true external performance [10, 64]. Prospective validation in independent cohorts using prespecified thresholds is required before these characteristics can be regarded as externally portable. For the same reason, optimal thresholds reported here are presented as descriptive study-level summaries rather than meta-analytically pooled cutoffs, as no validated framework exists for pooling Youden-derived thresholds across heterogeneous measurement protocols. The descriptive study-level reference values reported here are intended as hypothesis-generating reference points and should not be applied as universal decision cutoffs without prospective validation in independent cohorts.

In addition, reference standard heterogeneity is an important limitation. Approximately 45% of included studies adjudicated PSL on plain radiographs alone, 34% on CT alone, and the remainder on combined or MRI. CT offers greater sensitivity for early peri-screw lucency [37] and plain radiographs are more susceptible to projection-related false negatives. Reference modality is also non-randomly distributed across surgical era, geography, and the index test, precluding meaningful modality-stratified analysis. Pooled estimates therefore reflect a composite radiographic endpoint rather than uniform discrimination.

Finally, evidence linking paraspinal muscle characteristics to PSL remains limited. Muscle analyses were derived from a small number of studies with heterogeneous segmentation methods, vertebral levels, and normalization strategies, and most did not account for concurrent bone quality or construct factors. As a result, muscle metrics should be interpreted as exploratory markers of vulnerability rather than independent predictors of fixation failure. This caution applies most directly to relative cross-sectional area metrics, where pooled estimates aggregate measurements normalized variably to intervertebral disc area or vertebral body area, and to multifidus, where segment-specific degenerative behavior means that level-of-measurement heterogeneity may obscure or distort true effects. Pooled muscle effect estimates should therefore be interpreted as directional rather than quantitatively portable.

Future research should focus on prospective, multicenter cohorts with standardized loosening definitions, prespecified imaging time points, and harmonized protocols for bone and muscle assessment. In particular, studies incorporating concurrent measurement of bone quality, muscle reserve, and construct-level variables within the same population are needed to clarify their relative and interactive contributions. The development and external validation of multivariable models that integrate substrate metrics with surgical parameters will be essential to move beyond single-metric risk stratification and toward clinically actionable, context-sensitive decision support.

## Conclusion

Across included studies, CT- and MRI-derived bone quality metrics, including Hounsfield units, VBQ, CT-BMD, and DEXA T-scores, demonstrated consistent associations with radiographic PSL after degenerative lumbar fusion. Diagnostic performance for Hounsfield units, VBQ, and CT-BMD was moderate and broadly comparable, supporting their use as pragmatic preoperative risk stratifiers rather than definitive diagnostic tests. Limited and heterogeneous evidence also suggests that reduced psoas muscle size may act as a complementary marker of vulnerability in selected cohorts. Collectively, these findings support the integration of opportunistic imaging metrics into preoperative planning to inform interface-protective strategies and construct optimization, while underscoring the need for prospective, standardized validation and multivariable modeling to define clinically portable thresholds and link risk stratification to outcome-modifying interventions. Importantly, no included study demonstrated that altering management on the basis of Hounsfield units, VBQ, CT-BMD, or psoas CSA reduces the incidence of PSL; these metrics therefore support preoperative risk stratification and construct planning, while outcome-modifying intervention remains unproven.

## Supplementary Information

Below is the link to the electronic supplementary material.

ESM 1 Supplementary Material 1 (DOCX 8.12 MB)

## Data Availability

No datasets were generated or analysed during the current study.

## Abbreviations

*AUC*: Area Under the Curve
*CENTRAL*: Cochrane Central Register of Controlled Trials
*CI*: Confidence Interval
*CSA*: Cross-Sectional Area
*CSF*: Cerebrospinal Fluid
*CT*: Computed Tomography
*CT-BMD*: CT-Derived Bone Mineral Density
*DEXA*: Dual-Energy X-ray Absorptiometry
*dOR*: Diagnostic Odds Ratio
*FN*: False Negative
*FP*: False Positive
*MD*: Mean Difference
*MFI*: Muscle Fat Index
*MRI*: Magnetic Resonance Imaging
*NLR*: Negative Likelihood Ratio
*ODI*: Oswestry Disability Index
*PBQ*: Pedicle Bone Quality
*PLIF*: Posterior Lumbar Interbody Fusion
*PLR*: Positive Likelihood Ratio
*PRISMA*: Preferred Reporting Items for Systematic Reviews and Meta-Analyses
*PROSPERO*: The International Prospective Register of Systematic Reviews
*PSL*: Pedicle Screw Loosening
*QUADAS-2*: Quality Assessment of Diagnostic Accuracy Studies 2
*QUIPS*: Quality in Prognosis Studies
*rfCSA*: Relative Functional Cross-Sectional Area
*rtCSA*: Relative Total Cross-Sectional Area
*ROI*: Region of Interest
*SMD*: Standardized Mean Difference
*SROC*: Summary Receiver Operating Characteristic
*TLIF*: Transforaminal Lumbar Interbody Fusion
*TN*: True Negative
*TP*: True Positive
*VAS*: Visual Analogue Scale
*VBQ*: Vertebral Bone Quality.
