# Supplementary material for: Pedicle screw loosening after degenerative lumbar fusion: a systematic review and meta-analysis of preoperative imaging-derived bone quality and paraspinal muscle metrics
Source: Acta Neurochir (Wien). 2026 Jul 30;168(1):249. doi: 10.1007/s00701-026-06984-3 (PMC13714044; doi:10.1007/s00701-026-06984-3)
Supplement: Supplementary file 1 [file 701_2026_6984_MOESM1_ESM.docx]

| **Database** | **Search Strategy** |
| --- | --- |
| PubMed  (n=174) | ("screw loosening"[tiab] OR "pedicle screw loosening"[tiab] OR "implant loosening"[tiab] OR "pedicle screw loos*"[tiab] OR loosening[tiab] OR "screw halo"[tiab] OR "radiolucent halo"[tiab])  AND  (lumbar[tiab] OR lumbosacral[tiab] OR "lumbar vertebrae"[Mesh])  AND  ("spinal fusion"[Mesh] OR "spinal fusion"[tiab] OR "lumbar fusion"[tiab] OR "interbody fusion"[tiab] OR TLIF[tiab] OR PLIF[tiab] OR ALIF[tiab] OR LLIF[tiab] OR OLIF[tiab] OR XLIF[tiab] OR PLF[tiab] OR "posterolateral fusion"[tiab] OR "transforaminal lumbar interbody fusion"[tiab] OR "posterior lumbar interbody fusion"[tiab] OR "anterior lumbar interbody fusion"[tiab] OR "lateral lumbar interbody fusion"[tiab] OR "oblique lumbar interbody fusion"[tiab])  AND  (sarcopenia[Mesh] OR osteoporosis[Mesh] OR "Bone Density"[Mesh] OR sarcopenia[tiab] OR sarcopaenia[tiab] OR "paraspinal muscle*"[tiab] OR "muscle mass"[tiab] OR "muscle density"[tiab] OR osteoporosis[tiab] OR "bone mineral density"[tiab] OR "bone density"[tiab] OR "vertebral bone density"[tiab] OR "vertebral bone quality"[tiab] OR VBQ[tiab] OR "Hounsfield unit*"[tiab] OR HU[tiab]) |
| Embase  (n=357) | ('screw loosening':ti,ab OR 'pedicle screw loosening':ti,ab OR 'implant loosening':ti,ab OR 'pedicle screw loos*':ti,ab OR loosening:ti,ab OR 'screw halo':ti,ab OR 'radiolucent halo':ti,ab)  AND  (lumbar:ti,ab OR lumbosacral:ti,ab OR 'lumbar vertebrae'/exp)  AND  ('spinal fusion'/exp OR 'spinal fusion':ti,ab OR 'lumbar fusion':ti,ab OR 'interbody fusion':ti,ab OR TLIF:ti,ab OR PLIF:ti,ab OR ALIF:ti,ab OR LLIF:ti,ab OR OLIF:ti,ab OR XLIF:ti,ab OR PLF:ti,ab OR 'posterolateral fusion':ti,ab OR 'transforaminal lumbar interbody fusion':ti,ab OR 'posterior lumbar interbody fusion':ti,ab OR 'anterior lumbar interbody fusion':ti,ab OR 'lateral lumbar interbody fusion':ti,ab OR 'oblique lumbar interbody fusion':ti,ab)  AND  (sarcopenia/exp OR osteoporosis/exp OR 'bone density'/exp OR sarcopenia:ti,ab OR sarcopaenia:ti,ab OR 'paraspinal muscle*':ti,ab OR 'muscle mass':ti,ab OR 'muscle density':ti,ab OR osteoporosis:ti,ab OR 'bone mineral density':ti,ab OR 'bone density':ti,ab OR 'vertebral bone density':ti,ab OR 'vertebral bone quality':ti,ab OR VBQ:ti,ab OR 'Hounsfield unit*':ti,ab OR HU:ti,ab) |
| CENTRAL  (n=0) | ("screw loosening" OR "pedicle screw loosening" OR "implant loosening" OR "pedicle screw loos*" OR loosening OR "screw halo" OR "radiolucent halo")  AND  (lumbar OR lumbosacral)  AND  ("spinal fusion" OR "lumbar fusion" OR "interbody fusion" OR TLIF OR PLIF OR ALIF OR LLIF OR OLIF OR XLIF OR PLF OR "posterolateral fusion" OR "transforaminal lumbar interbody fusion" OR "posterior lumbar interbody fusion" OR "anterior lumbar interbody fusion" OR "lateral lumbar interbody fusion" OR "oblique lumbar interbody fusion")  AND  (sarcopenia OR sarcopaenia OR "paraspinal muscle*" OR "muscle mass" OR "muscle density" OR osteoporosis OR "bone mineral density" OR "bone density" OR "vertebral bone density" OR "vertebral bone quality" OR VBQ OR "Hounsfield unit*" OR HU) |

**Supplementary Table 1*:* Search strategies**

Full electronic search strategies used to identify relevant studies for this review, reported for each database. n = number of articles returned.

**Supplementary Table 2: Risk of bias assessment**

Summary of risk of bias assessment for diagnostic accuracy studies (QUADAS-2), reported by individual domain and overall risk of bias.

**Supplementary Table 3: Certainty of evidence (GRADE)**

GRADE assessment of certainty of evidence for each primary diagnostic metric in the present review.

**Question**: Should Hounsfield units be used to diagnose screw loosening in patients receiving lumbar fusion?

| Sensitivity | 0.81 (95% CI: 0.72 to 0.87) |
| --- | --- |
| Specificity | 0.60 (95% CI: 0.55 to 0.64) |

| Prevalence | 20% |
| --- | --- |

| Outcome | № of studies (№ of patients) | Study design | Factors that may decrease certainty of evidence | | | | | Effect per 1,000 patients tested | Test accuracy CoE | Overall downgrading | | |
| --- | --- | --- | --- | --- | --- | --- | --- | --- | --- | --- | --- | --- |
|  |  |  | Risk of bias | Indirectness | Inconsistency | Imprecision | Publication bias | pre-test probability of 20% |  |  |  |  |
| **True positives** | 6 studies 212 patients | cohort & case-control type studies | serious^a^ [-1] | not serious  [-0] | not serious  [-0] | not serious  [-0] | none  [-0] | 162 (144 to 174) | ⨁⨁⨁◯ Moderate^a^ | | | [-1]  (risk of bias) |
| **False negatives** |  |  |  |  |  |  |  | 38 (26 to 56) |  |  |  |  |
| **True negatives** | 6 studies 631 patients | cohort & case-control type studies | serious^a^  [-1] | not serious  [-0] | not serious  [-0] | not serious  [-0] | none  [-0] | 480 (440 to 512) | ⨁⨁⨁◯ Moderate^a^ | | [-1]  (risk of bias) | |
| **False positives** |  |  |  |  |  |  |  | 320 (288 to 360) |  |  |  |  |

a. 6 'moderate' on QUADAS-2 risk of bias

**Supplementary Table 3A: Hounsfield units.**

**Question**: Should VBQ be used to diagnose screw loosening in patients receiving lumbar fusion?

| Sensitivity | 0.83 (95% CI: 0.78 to 0.87) |
| --- | --- |
| Specificity | 0.68 (95% CI: 0.61 to 0.74) |

| Prevalence | 20% |
| --- | --- |

| Outcome | № of studies (№ of patients) | Study design | Factors that may decrease certainty of evidence | | | | | Effect per 1,000 patients tested | Test accuracy CoE | | Overall downgrading |
| --- | --- | --- | --- | --- | --- | --- | --- | --- | --- | --- | --- |
|  |  |  | Risk of bias | Indirectness | Inconsistency | Imprecision | Publication bias | pre-test probability of 20% |  |  |  |
| **True positives** | 8 studies 387 patients | cohort & case-control type studies | serious^a^  [-1] | not serious  [-0] | not serious  [-0] | not serious  [-0] | none  [-0] | 166 (156 to 174) | ⨁⨁⨁◯ Moderate^a^ | | [-1]  (risk of bias) |
| **False negatives** |  |  |  |  |  |  |  | 34 (26 to 44) |  |  |  |
| **True negatives** | 8 studies 1162 patients | cohort & case-control type studies | serious^a^  [-1] | not serious  [-0] | not serious  [-0] | not serious  [-0] | none  [-0] | 544 (488 to 592) | ⨁⨁⨁◯ Moderate^a^ | [-1]  (risk of bias) | |
| **False positives** |  |  |  |  |  |  |  | 256 (208 to 312) |  |  |  |

a. 8 'moderate' on QUADAS-2 risk of bias

**Supplementary Table 3B: Vertebral bone quality (VBQ)**

**Question**: Should BMD be used to diagnose screw loosening in patients receiving lumbar fusion?

| Sensitivity | 0.83 (95% CI: 0.75 to 0.89) |
| --- | --- |
| Specificity | 0.62 (95% CI: 0.46 to 0.76) |

| Prevalence | 20% |
| --- | --- |

| Outcome | № of studies (№ of patients) | Study design | Factors that may decrease certainty of evidence | | | | | Effect per 1,000 patients tested | Test accuracy CoE | Overall downgrading |
| --- | --- | --- | --- | --- | --- | --- | --- | --- | --- | --- |
|  |  |  | Risk of bias | Indirectness | Inconsistency | Imprecision | Publication bias | pre-test probability of 20% |  |  |
| **True positives** | 3 studies 113 patients | cohort & case-control type studies | serious^a^  [-1] | not serious  [-0] | not serious  [-0] | not serious  [-0] | none  [-0] | 166 (150 to 178) | ⨁⨁⨁◯ Moderate^a^ | [-1]  (risk of bias) |
| **False negatives** |  |  |  |  |  |  |  | 34 (22 to 50) |  |  |
| **True negatives** | 3 studies 284 patients | cohort & case-control type studies | serious^a^  [-1] | not serious  [-0] | not serious  [-0] | not serious  [-0] | none  [-0] | 496 (368 to 608) | ⨁⨁⨁◯ Moderate^a^ | [-1]  (risk of bias) |
| **False positives** |  |  |  |  |  |  |  | 304 (192 to 432) |  |  |

a. 3 'moderate' on QUADAS-2 risk of bias

**Supplementary Table 3C: CT- Bone mineral density (CT-BMD)**

**Supplementary Figure 1: T-score**

Forest plots (random effects model) comparing T-scores between patients with (‘SL’) and without screw loosening (‘No SL’). Individual study estimates are shown as squares proportional to study weight. Horizontal lines indicate 95% confidence intervals. Diamond represents pooled estimate for overall effect. IV = inverse variance; MD = mean difference.

**Supplementary Figure 2: AUC for Hounsfield units**

Forest plot (random effects model) reporting area under the receiver operating characteristic curve (AUC) for Hounsfield units in predicting screw loosening. Individual study estimates are shown as squares proportional to study weight. Horizontal lines indicate 95% confidence intervals. Diamond represents pooled estimate for AUC. HK = Hartung-Knapp.

**Supplementary Figure 3: AUC for VBQ**

Forest plot (random effects model) reporting area under the receiver operating characteristic curve (AUC) for vertebral bone quality (VBQ) in predicting screw loosening. Individual study estimates are shown as squares proportional to study weight. Horizontal lines indicate 95% confidence intervals. Diamond represents pooled estimate for AUC. HK = Hartung-Knapp.

**Supplementary Figure 4: AUC for CT-BMD**

Forest plot (random effects model) reporting area under the receiver operating characteristic curve (AUC) for CT-BMD in predicting screw loosening. Individual study estimates are shown as squares proportional to study weight. Horizontal lines indicate 95% confidence intervals. Diamond represents pooled estimate for AUC. HK = Hartung-Knapp.

**Supplementary Figure 5: Psoas muscle rtCSA**

Forest plot (random effects model) reporting pooled comparisons of psoas muscle relative total cross-sectional area (rtCSA) between patients with (SL) and without screw loosening (No SL). Individual study estimates are shown as squares proportional to study weight. Horizontal lines indicate 95% confidence intervals. Diamond represents pooled estimate for effect. Negative values indicate lower psoas rtCSA in the SL group.

**Supplementary Figure 6: Psoas muscle rfCSA**

Forest plot (random effects model) reporting pooled comparisons of psoas muscle relative functional cross-sectional area (rfCSA) between patients with (SL) and without screw loosening (No SL). Individual study estimates are shown as squares proportional to study weight. Horizontal lines indicate 95% confidence intervals. Diamond represents pooled estimate for effect. Negative values indicate lower psoas rfCSA in the SL group.

**Supplementary Figure 7: Psoas muscle MFI**

Forest plot (random effects model) reporting pooled comparisons of psoas muscle-fat index (MFI) between patients with (SL) and without screw loosening (No SL). Individual study estimates are shown as squares proportional to study weight. Horizontal lines indicate 95% confidence intervals. Diamond represents pooled estimate for effect. Negative values indicate lower psoas MFI in the SL group.

**Supplementary Figure 8: Erector spinae rtCSA**

Forest plot (random effects model) reporting pooled comparisons of erector spinae relative total cross-sectional area (rtCSA) between patients with (SL) and without screw loosening (No SL). Individual study estimates are shown as squares proportional to study weight. Horizontal lines indicate 95% confidence intervals. Diamond represents pooled estimate for effect. Negative values indicate lower erector spinae rtCSA in the SL group.

**Supplementary Figure 9: Erector spinae rfCSA**

Forest plot (random effects model) reporting pooled comparisons of erector spinae relative functional cross-sectional area (rfCSA) between patients with (SL) and without screw loosening (No SL). Individual study estimates are shown as squares proportional to study weight. Horizontal lines indicate 95% confidence intervals. Diamond represents pooled estimate for effect. Negative values indicate lower erector spinae rfCSA in the SL group.

**Supplementary Figure 10: Erector spinae MFI**

Forest plot (random effects model) reporting pooled comparisons of erector spinae muscle-fat index (MFI) between patients with (SL) and without screw loosening (No SL). Individual study estimates are shown as squares proportional to study weight. Horizontal lines indicate 95% confidence intervals. Diamond represents pooled estimate for effect. Negative values indicate lower erector spinae MFI in the SL group.

**Supplementary Figure 11: Multifidus rtCSA**

Forest plot (random effects model) reporting pooled comparisons of multifidus relative total cross-sectional area (rtCSA) between patients with (SL) and without screw loosening (No SL). Individual study estimates are shown as squares proportional to study weight. Horizontal lines indicate 95% confidence intervals. Diamond represents pooled estimate for effect. Negative values indicate lower multifidus rtCSA in the SL group.

**Supplementary Figure 12: Multifidus rfCSA**

Forest plot (random effects model) reporting pooled comparisons of multifidus relative functional cross-sectional area (rfCSA) between patients with (SL) and without screw loosening (No SL). Individual study estimates are shown as squares proportional to study weight. Horizontal lines indicate 95% confidence intervals. Diamond represents pooled estimate for effect. Negative values indicate lower multifidus rfCSA in the SL group.

**Supplementary Figure 13: Multifidus MFI**

Forest plot (random effects model) reporting pooled comparisons of multifidus muscle-fat index (MFI) between patients with (SL) and without screw loosening (No SL). Individual study estimates are shown as squares proportional to study weight. Horizontal lines indicate 95% confidence intervals. Diamond represents pooled estimate for effect. Negative values indicate lower multifidus MFI in the SL group.

**Supplementary Figure 14: Fusion**

Forest plots (random effects model) comparing fusion rate (%) between patients with (‘SL’) and without screw loosening (‘No SL’). Individual study estimates are shown as squares proportional to study weight. Horizontal lines indicate 95% confidence intervals. Diamond represents pooled estimate for overall effect. IV = inverse variance; RR = risk ratio.

**A**

**D**

**C**

**B**

**Supplementary Figure 15: Pain and disability scores**

Forest plots (random effects model) comparing (A) preoperative ODI, (B) postoperative ODI, (C) preoperative VAS, (D) postoperative VAS between patients with screw loosening (‘SL’) and without screw loosening (‘No SL’). Individual study estimates are shown as squares proportional to study weight. Horizontal lines indicate 95% confidence intervals. Diamond represents pooled estimate for overall effect. IV = inverse variance; MD = mean difference.

**B**

**A**

**D**

**C**

**E**

**F**

**Supplementary Figure 16. Baujat plots for selected pairwise meta-analyses.**

(A) CT-derived bone mineral density (CT-BMD). (B) CT Hounsfield unit (HU). (C) Fusion. (D) MRI-based vertebral bone quality (VBQ). (E) Dual-energy x-ray absorptiometry T-score. (F) Preoperative visual analog scale (VAS) back pain. The x-axis shows each study’s contribution to overall heterogeneity, and the y-axis shows its influence on the pooled effect estimate. These plots were used to identify influential studies and the main contributors to between-study heterogeneity.

**Supplementary Figure 17: Publication bias assessment**

Deeks’ funnel plots for assessment of publication bias for primary outcome Hounsfield units. Diagnostic odds ratio is plotted against the inverse square root of the effective sample size. Circles represent individual studies, and the dashed line denotes the weighted linear regression line used in Deeks’ asymmetry test.
